# Phase 1/2a trial of intravenous BAL101553, a novel controller of the spindle assembly checkpoint, in advanced solid tumours

**DOI:** 10.1038/s41416-020-1010-8

**Published:** 2020-08-03

**Authors:** Rebecca Kristeleit, Jeffry Evans, L. Rhoda Molife, Nina Tunariu, Heather Shaw, Sarah Slater, Noor R. Md Haris, Nicholas F. Brown, Martin D. Forster, Nikolaos Diamantis, Robert Rulach, Alastair Greystoke, Uzma Asghar, Mihaela Rata, Stephanie Anderson, Felix Bachmann, Alison Hannah, Thomas Kaindl, Heidi A. Lane, Patrice J. Larger, Anne Schmitt-Hoffmann, Marc Engelhardt, Alexandar Tzankov, Ruth Plummer, Juanita Lopez

**Affiliations:** 1 Department of Oncology, Guys and St Thomas‘ NHS Foundation Trust, London, UK; 2 National Institute for Health Research, University College London Hospitals Clinical Research Facility, London, UK; 3 University of Glasgow, Glasgow, UK; 4 The Beatson West of Scotland Cancer Centre, Glasgow, UK; 5 Drug Development Unit, The Royal Marsden Hospital and the Institute of Cancer Research, Sutton, UK; 6 Sir Bobby Robson Cancer Trials Research Centre, Northern Centre for Cancer Care, Freeman Hospital, Newcastle upon Tyne, UK; 7 Newcastle University, Newcastle upon Tyne, UK; 8 Radiotherapy and Imaging Unit, Institute of Cancer Research and Royal Marsden Hospital, Sutton, UK; 9 Basilea Pharmaceutica International Ltd, Basel, Switzerland; 10 Oncology Clinical Trial Consulting, Sebastopol, CA USA; 11 Institute of Medical Genetics and Pathology, Basel, Switzerland

**Keywords:** Cancer, Molecular medicine

## Abstract

**Background:**

BAL101553 (lisavanbulin), the lysine prodrug of BAL27862 (avanbulin), exhibits broad anti-proliferative activity in human cancer models refractory to clinically relevant microtubule-targeting agents.

**Methods:**

This two-part, open-label, phase 1/2a study aimed to determine the maximum tolerated dose (MTD) and dose-limiting toxicities (DLTs) of 2-h infusion of BAL101553 in adults with advanced or recurrent solid tumours. The MTD was determined using a modified accelerated titration design in phase I. Patients received BAL101553 at the MTD and at lower doses in the phase 2a expansion to characterise safety and efficacy and to determine the recommended phase 2 dose (RP2D).

**Results:**

Seventy-three patients received BAL101553 at doses of 15–80 mg/m^2^ (phase 1, *n* = 24; phase 2a, *n* = 49). The MTD was 60 mg/m^2^; DLTs observed at doses ≥60 mg/m^2^ were reversible Grade 2–3 gait disturbance with Grade 2 peripheral sensory neuropathy. In phase 2a, asymptomatic myocardial injury was observed at doses ≥45 mg/m^2^. The RP2D for 2-h intravenous infusion was 30 mg/m^2^. The overall disease control rate was 26.3% in the efficacy population.

**Conclusions:**

The RP2D for 2-h infusion of BAL101553 was well tolerated. Dose-limiting neurological and myocardial side effects were consistent with the agent’s vascular-disrupting properties.

**Clinical trial registration:**

EudraCT: 2010-024237-23.

## Background

Microtubules are present in both interphase- and dividing cells and are involved in several critical cellular functions, including mitosis, intracellular trafficking, cell signalling, migration, secretion, and angiogenesis.^1–3^ Microtubule-targeting agents (MTAs) can disrupt microtubule and mitotic spindle function, with subsequent anti-tumour and vascular-disrupting effects. Conventional MTAs fall into two main groups: microtubule-destabilising agents (including *Vinca* alkaloids, halichondrins, and combretastatins) that destabilise or depolymerise microtubules, and microtubule-stabilising agents (including taxanes and epothilones) that polymerise microtubules.^3,4^ At the cellular level, both groups of agents suppress spindle-microtubule dynamics, causing mitotic arrest, and trigger cell death through apoptosis.^3^

Microtubule-targeting agents are among the most active cytotoxic anti-cancer drugs currently in use. However, despite a high initial sensitivity of many malignancies to MTAs, resistance invariably arises through several mechanisms that are postulated to include drug efflux pump (P-glycoprotein) overexpression, aberrant expression of tubulin isotypes and *BRCA1*, and deregulated cell survival pathways.^3,5–7^ There is an urgent clinical need for novel treatment options for patients with advanced solid tumours, who are refractory to conventional MTAs.

BAL101553 (lisavanbulin) is a water-soluble lysine prodrug of the active furazano-benzimidazole derivative BAL27862 (avanbulin), a novel, synthetic molecule that shows promising anti-tumour activity.^8,9^ Non-clinical studies have revealed that BAL27862 acts by destabilising microtubules through a unique mechanism of action distinct from that of other MTAs, arresting tumour cell proliferation in the G2/M phase of the cell cycle and inducing apoptosis.^9,10^ BAL27862 shares the intradimer interface, a tubulin binding site, with colchicine and disrupts microtubule organisation thereby inducing the formation of the ‘spindle assembly checkpoint’.^9,10^

BAL101553 has demonstrated broad anti-tumour activity across a panel of cell lines and xenograft models.^11–20^ Importantly, BAL101553 is active in human cancer models that are refractory to standard of care therapeutics, including clinically relevant MTAs, due to P-glycoprotein overexpression and non-P-glycoprotein-related mechanisms.^14,15,20^ Hence, the activity of BAL101553 does not appear to be affected by factors that confer resistance to conventional MTAs. BAL101553 also targets the tumour microenvironment^14^ causing tumour vascular disruption,^21^ which has also been described for other MTAs. BAL101553 can be administered intravenously or orally.

Here, we report results from the first-in-human dose-escalation study of BAL101553 monotherapy in patients with advanced cancer.

## Methods

### Study design

This was a two-part, open-label, phase 1/2a study of single-agent BAL101553 in patients with advanced solid tumours. The study was conducted in accordance with the Declaration of Helsinki and Good Clinical Practice. Institutional Review Boards at the four study sites in the UK approved the study and all participants provided written informed consent.

The primary objectives were to determine the maximum tolerated dose (MTD) and characterise the dose-limiting toxicities (DLTs) of single-agent BAL101553 administered intravenously over 2 h on days 1, 8, and 15 of a 28-day treatment cycle. Secondary objectives comprised evaluation of the safety and tolerability of BAL101553, the pharmacokinetics (PK) of BAL101553 and BAL27862, and the anti-tumour activity of BAL101553. Exploratory objectives were to characterise the pharmacodynamic effects of BAL101553/BAL27862, including functional vascular imaging, and to explore the potential utility of biomarkers in blood and/or tumour tissue for patient stratification.

The MTD of BAL101553 was determined by dose-escalation in the phase 1 part of the study. The phase 2a expansion part was originally intended to characterise the safety, tolerability, and efficacy of BAL101553 at the MTD level determined in phase 1. Based on emergent data during the study, the design of the phase 2a part was modified in several amendments to investigate whether a lower dose level was clinically preferable to dosing at the MTD. The design was changed from a fixed-dose treatment of 20 patients at the MTD to an open-label randomised (1:1) design that randomised 40 patients to receive either the MTD or 50% of the MTD. Randomisation of patients during this period of the study is described in Supplementary Text 1. In subsequent amendments the tested doses were further reduced and ultimately all remaining patients were treated at the 50% MTD dose level.

### Patients

Eligible patients were aged 18 years or older with advanced or recurrent solid tumours who had failed standard therapy, or for whom no standard therapy was available. Patients who had received previous treatment with taxanes or other MTAs were permitted. In the phase 2a part of the study, inclusion was limited to patients with colorectal, gastric, or gastro-oesophageal junction, non-small-cell lung, ovarian (including primary peritoneal), pancreatic (including ampullary), or triple-negative breast cancers to limit variability. These six cancer types were selected based on clinical and/or pharmacodynamic efficacy signals in the phase 1 part of the study, and biomarker studies of human tumour tissue libraries suggested that these cancers typically express biomarkers considered relevant for the anti-tumour efficacy of BAL101553, i.e., microtubule- and spindle assembly checkpoint-related markers (such as BubR1).

BuBR1 staining was performed in available tissues from patients participating in this study. However, these were exploratory studies and not performed as a selection biomarker prior to study entry (data not shown).

All patients had measurable disease according to RECIST (Response Evaluation Criteria In Solid Tumours) criteria v1.1 documented within 35 days prior to starting study drug: an Eastern Cooperative Oncology Group (ECOG) performance status ≤ 1; adequate organ and marrow function; and a life expectancy of ≥12 weeks.

Patients with peripheral neuropathy Grade ≥ 2 (National Cancer Institute Common Terminology Criteria for Adverse Events [NCI-CTCAE] v4.03); systolic blood pressure ≥ 140 mmHg and/or diastolic BP ≥ 90 mmHg; significant cardiac or cerebrovascular disease; those treated with a calcium channel blocker, or who required a combination of more than two antihypertensive medications to control blood pressure and patients requiring vitamin K antagonists were excluded from study entry. Full inclusion/exclusion criteria are listed in Supplementary Text 2.

### Study treatment

BAL101553 was administered intravenously over 2 h on days 1, 8, and 15 of a 28-day cycle. Intravenous dosing was used as the oral formulation of BAL101553 was not available at the time of this study. Dosing on days 1, 8, and 15 over a 28-day cycle was chosen based on the expected gastrointestinal and haematological toxicity (observed in 3-week Good Laboratory Practice toxicity studies in rats and dogs with weekly intravenous administration), with the goal to have a 14-day drug-free interval to recover from such toxicity. Treatment was continued until disease progression, unacceptable toxicity or until investigator/patient decision to withdraw.

Intra-patient dose escalation to a higher dose already considered safe was allowed in the phase 1 part of the study for patients who had completed two or more cycles of BAL101553 without any Grade ≥ 2 drug-related adverse events (AEs). Dose interruptions were allowed for a DLT until recovery to CTCAE Grade ≤ 1 or baseline; subsequent doses of BAL101553 were reduced by one dose level. Dose interruptions and reductions were also allowed for non-DLT events. For cycle 1, if a patient did not meet the requirements for being dosed on either day 8 or day 15, the physician could delay treatment for up to 5 days. BAL101553 was administered if the patient met re-treatment criteria within these 5 days. Cycle 1 was regarded as complete if all three doses of BAL101553 were administered within 28 days, with recovery of any toxicity to permit initiation of cycle 2 with a maximum delay of 14 days.

#### Dose escalation

The starting weekly dose of BAL101553 (15 mg/m^2^) was based on Good Laboratory Practice toxicology studies in dogs and was approximately 1/6th of the highest non-severely toxic dose observed in these studies. This corresponded to a dose of approximately 27.5 mg of BAL101553 for a 1.73 m^2^, 70-kg patient.

Full details of the dosing criteria are presented in Supplementary Table 1. Dose escalation in the Phase 1 part of the study was performed using a modified accelerated 3 + 3 titration design. Patients were enrolled in sequential dose cohorts comprising one to six patients; cohorts were expanded if a patient experienced a BAL101553 treatment-related DLT during cycle 1. DLTs were generally defined as Grade ≥ 4 haematological AEs or Grade ≥ 3 non-haematological AEs (full DLT criteria are defined in Supplementary Text 3).

Dose cohort escalation decisions incorporated clinical review of all relevant available data from contemporaneous and previous dose cohorts. The maximum administered dose was defined as the dose level at which a DLT was observed during treatment cycle 1 in ≥33% of evaluable patients. The MTD was defined as the highest dose level below the maximum administered dose with an acceptable tolerability profile. The recommended phase 2 dose (RP2D) was determined based on all available safety, PK, pharmacodynamic, and efficacy data.

### Study assessments

#### Safety

Safety was assessed throughout the study. Assessments included recording of all AEs (according to NCI-CTCAE v4.03) and serious AEs (SAEs), laboratory parameters, echocardiography, vital signs, ECOG performance status, physical examination, radiology assessments, and evaluation of concomitant medications.

#### Pharmacokinetics

Blood samples for assessment of BAL101553 and BAL27862 pharmacokinetics were taken from patients in phase 1 of the study on day 1 of cycles 1 and 2 (pre-dose, and at 1, 2 [immediately prior to end of infusion], 2.5, 3, 4, 6, 8, 24, and 48 h after the start of the 2-h infusion). In the phase 2a part of the study, samples were taken on day 1 of cycle 1 (pre-dose, 2 [immediately prior to end of infusion], 4, 6, 10, and 24 h after the start of the 2-h infusion). Additional samples were taken immediately prior to the end of the 2-h infusion on days 8 and 15 of cycle 1 (and cycle 2 in phase 1), at the occurrence of a DLT, and from patients undergoing intra-patient dose escalation or dose reduction. Urine samples were taken on day 1 of cycles 1 and 2 from patients in the phase 1 part of the study for calculation of total 24-h urinary excretion of BAL101553 and BAL27862. BAL101553 and BAL27862 were quantified in plasma and urine using liquid chromatography tandem-mass spectrometry with a lower limit of quantification of 1 ng/mL.

#### Pharmacodynamics

Blood samples obtained on days 1, 15, and 22 of cycle 1, and day 22 of cycle 2 were evaluated for circulating tumour cell, circulating endothelial cell, and circulating endothelial progenitor cell counts. Tumour biopsies (where possible) were to be taken at screening, on day 22 of cycles 1, 2, and one subsequent cycle, and at disease progression. Tumour biopsies were analysed by immunohistochemistry for exploratory patient biomarkers. In phase 2a, serial dynamic contrast-enhanced magnetic resonance imaging (MRI) and diffusion-weighted MRI were conducted in suitable patients to assess the effect of BAL101553 on tumour vascularity and cellularity. Imaging was conducted at two pre-treatment visits (2–3 days apart) and three post-treatment visits (days 1, 2, and 8 of cycle 1).

#### Efficacy

Tumour response was assessed using computed tomography scans according to RECIST v1.1 every two cycles (8 weeks) in patients with measurable disease.

### Statistical analyses

#### Study populations

The modified accelerated 3 + 3 design and the exploratory nature of the phase 2a expansion portion did not require sample size calculation justification.

Patients eligible for determining the MTD had to have received all three doses of BAL101553 during cycle 1 (or at least one dose if the patient experienced a DLT) and been followed for ≥28 days after the first dose for safety (MTD population). Safety was evaluated in all recruited patients (safety population). Pharmacokinetics were evaluated in all patients who received at least one partial or complete dose of BAL101553 and had at least one post-baseline PK assessment (PK population). Anti-tumour activity was evaluated in all patients who received all three doses of BAL101553 in cycle 1 and who either underwent at least one on-study tumour assessment or a radiological assessment by RECIST guidelines at or after the end of cycle 2, or who showed clinical and/or radiological progressive disease prior to the end of cycle 2 (efficacy population). Efficacy parameters were also analysed using the full analysis population (all patients who received at least one partial or complete dose of BAL101553 based on the intent-to-treat principle).

#### Statistical assessments

Demographic, baseline, safety, PK, and pharmacodynamic data were analysed by dose cohort using descriptive statistics. Pharmacokinetic parameters were calculated from plasma and urine concentration data by non-compartmental analysis using Phoenix WinNonLin 7.0. Anti-tumour activity was assessed by dose cohort summarising the objective response rate (ORR), disease control rate (DCR), and progression-free survival (PFS) with exact 95% confidence intervals (CI). The ORR was defined as complete response plus partial response (PR); the DCR was defined as complete response plus PR plus stable disease (SD) lasting two or more cycles from start of BAL101553 treatment to earliest date of clinical/objective progression. Progression-free survival was defined as the interval between the date of first infusion and the earliest date of clinical/objective progression or death. Median time to progression or death was also determined by the Kaplan−Meier method.

## Results

### Patient demographics and disposition

Seventy-three patients were enrolled at four sites in the UK. The first patient, first visit was on 12 July 2011 and the last patient, last visit was on 6 April 2016. Baseline demographic and disease data are presented in Table 1 and by dose group in Supplementary Table 2. Overall, 53.4% of patients were male, 90.4% were white Caucasian and median age was 59 years (range 29–80). ECOG performance status at baseline was 0 for 23 (31.5%) patients and 1 for 50 (68.5%) patients. The most prevalent cancer types were colorectal cancer (30.1%), non-small-cell lung cancer (12.3%), and pancreatic/ampullary cancer (12.3%). Thirty-four (46%) patients had previously been treated with MTAs, of which 24 had discontinued treatment due to documented disease progression.

**Table 1:** Baseline demographic and disease history data of the 73 enrolled and treated patients.

	Total (*n* = 73)
Age (years), median (range)	59.0 (29–80)
Female/Male, *n* (%)	34 (46.6)/39 (53.4)
ECOG PS 0/1, *n* (%)	23 (31.5)/50 (68.5)
Prior treatment regimens	
Overall, median (range)	3 (0–8)
Chemotherapy/hormone therapy, *n* (%)	72 (98.6)
Prior MTAs, *n* (%)	34 (46.6)
Radiotherapy, *n* (%)	29 (39.7)
Surgery, *n* (%)	52 (71.2)
Most common tumour types	
Colorectal	22 (30.1)
NSCLC	9 (12.3)
Pancreatic/ampullary cancer	9 (12.3)
Gastro-oesophageal cancer	8 (11.0)
Ovarian/primary peritoneal cancer	8 (11.0)
TNBC	4 (5.5)
Other^a^	13 (17.8)
Tumour histology, *n* (%)	
Adenocarcinoma	59 (80.8)
Squamous cell carcinoma	3 (4.1)
Other	11 (15.1)
Metastatic disease, *n* (%)	66 (91.7)
Histopathological grade, *n* (%)	
Grade 1	0
Grade 2	24 (33.8)
Grade 3	19 (26.8)
Not assessable	16 (22.5)
Other	12 (16.9)
Missing	2

*ECOG PS* Eastern Cooperative Oncology Group performance status, *MTA* microtubule-targeting agent, *NSCLC* non-small-cell lung cancer, *TNBC* triple-negative breast cancer.

^a^Other comprises: two cases of oesophageal cancer and single cases of adrenocortical cancer, anal cancer, cervical cancer, cholangiocellular cancer, epitheliod mesothelioma, gastric cancer, laryngeal cancer, mesenchymal chondrosarcoma, neuroendocrine cancer, small bowel cancer, thymoma.

### Phase 1

All patients were treated with at least one dose of BAL101553 at dose levels between 15 and 80 mg/m^2^. Twenty-four patients received BAL101553 at starting dose levels of 15 mg/m^2^ (*n* = 1), 30 mg/m^2^ (*n* = 3), 45 mg/m^2^ (*n* = 3), 60 mg/m^2^ (*n* = 10), and 80 mg/m^2^ (*n* = 7). Two of six evaluable patients treated at a starting dose of 80 mg/m^2^ experienced a DLT of reversible Grade 2–3 gait disturbance. The gait disturbance in both cases occurred with fully or partially reversible Grade 2 peripheral sensory neuropathy. The reduction in proprioception/sensation contributed to the gait disturbance. Therefore, the MTD for BAL101553 administered as a 2-h infusion on days 1, 8, and 15 of a 28-day cycle was determined to be 60 mg/m^2^ and the maximum administered dose was 80 mg/m^2^. One of the six evaluable patients treated at the MTD experienced a similar toxicity (Grade 3 reduced mobility with dizziness).

### Phase 2a

Following the changes in study design, patients were randomised to receive BAL101553 in the phase 2a part at either the MTD (60 mg/m^2^) or 50% of the MTD (30 mg/m^2^). Three additional AEs meeting the criteria for a DLT were reported in the phase 2a part of the study. These events necessitated that the maximum dose investigated in phase 2a was reduced to 45 mg/m^2^ after two patients treated at 60 mg/m^2^ experienced myocardial injury (Grade 3 troponin elevation and electrocardiogram changes including T-wave inversions). Subsequently, a further patient treated at 45 mg/m^2^ experienced asymptomatic myocardial infarction and the dose cohort level was lowered to 30 mg/m^2^. Overall, 49 patients received BAL101553 at starting dose levels of 30 mg/m^2^ (*n* = 33), 45 mg/m^2^ (*n* = 5), and 60 mg/m^2^ (*n* = 11) in the phase 2a expansion part. The RP2D for 2-h intravenous infusion of BAL101553 was determined to be 30 mg/m^2^.

### Overall safety data

The median duration of study-drug treatment was 43.0 days (range 1–1032) and two cycles (range 1–37). Four (5.5%) patients withdrew consent: two due to DLTs, one due to the time-intensive schedule of the trial, and one for whom no reason was given. Six (8.2%) patients died during the study or within 30 days following the last dose. One patient died during screening and was never dosed. The other five deaths occurred within 28 days of the last study drug administration; four were due to disease progression and judged not to be drug related. One patient (30 mg/m^2^ BAL101553) died due to a suspected unexpected serious adverse reaction of acute abdomen, which, although primarily attributed to cancer progression (gastro-oesophageal junction carcinoma with liver metastases), was assessed as possibly related to BAL101553.

Adverse events judged to be drug-related occurred in 65 (89.0%) patients (Table 2). The most common were nausea (43.8%), vomiting (34.2%), hypertension (32.9%), fatigue (31.5%), diarrhoea (30.1%), peripheral neuropathy (17.8%), and decreased appetite (17.8%). Twenty-two (30.1%) patients experienced 69 treatment-related and dose-dependent severe (Grade 3−4) events. Hypertension was the most common Grade 3−4 treatment-related AE, occurring in 18 (24.7%) patients. Five out of seven (71.4%) evaluable patients in the 80 mg/m^2^ cohort and 10 out of 21 (47.6%) patients in the 60 mg/m^2^ cohort experienced Grade 3/4 hypertension. Only one (2.8%) patient treated at the RP2D (30 mg/m^2^) experienced Grade 3 hypertension.

**Table 2:** Treatment-related adverse events occurring in >5% of the total population and all Grade 3–4 events.

System organ class/preferred term, *n* (%)	15 mg/m^2^ [*n* = 1]		30 mg/m^2^ [*n* = 36]		45 mg/m^2^ [*n* = 8]		60 mg/m^2^ [*n* = 21]		80 mg/m^2^ [*n* = 7]		Total [*n* = 73]	
	Grade 1−2	Grade 3−4	Grade 1−2	Grade 3−4	Grade 1−2	Grade 3−4	Grade 1−2	Grade 3−4	Grade 1−2	Grade 3−4	Grade 1−2	Grade 3−4
All BAL101553-related AEs	1 (100)	0	26 (72.2)	2 (5.6)	6 (75.0)	2 (25.0)	8 (38.1)	13 (61.9)	2 (28.6)	5 (71.4)	43 (58.9)	22 (30.1)
Gastrointestinal disorders	1 (100)	0	18 (50.0)	0	5 (62.5)	0	16 (76.2)	2 (9.5)	7 (100)	0	47 (64.4)	2 (2.7)
Nausea	1 (100)	0	11 (30.6)	0	2 (25.0)	0	12 (57.1)	0	6 (85.7)	0	32 (43.8)	0
Vomiting	1 (100)	0	3 (8.3)	0	2 (25.0)	0	12 (57.1)	0	7 (100)	0	25 (34.2)	0
Diarrhoea	0	0	5 (13.9)	0	4 (50.0)	0	9 (42.9)	0	4 (57.1)	0	22 (30.1)	0
Abdominal pain	0	0	3 (8.3)	0	0	0	2 (9.5)	1 (4.8)	1 (14.3)	0	6 (8.2)	1 (1.4)
Constipation	0	0	0	0	0	0	3 (14.3)	0	1 (14.3)	0	4 (5.5)	0
Abdominal pain upper	0	0	0	0	0	0	1 (4.8)	1 (4.8)	1 (14.3)	0	2 (2.7)	1 (1.4)
General disorders/administration site conditions	1 (100)	0	12 (33.3)	1 (2.8)	4 (50.0)	0	11 (52.4)	1 (4.8)	4 (57.1)	1 (14.3)	32 (43.8)	3 (4.1)
Fatigue	0	0	6 (16.7)	1 (2.8)	2 (25.0)	0	9 (42.9)	1 (4.8)	4 (57.1)	0	21 (28.8)	2 (2.7)
Pyrexia	0	0	2 (5.6)	0	1 (12.5)	0	4 (19.0)	0	2 (28.6)	0	9 (12.3)	0
Infusion site reaction	1 (100)	0	4 (11.1)	0	0	0	1 (4.8)	0	0	0	6 (8.2)	0
Chills	0	0	1 (2.8)	0	0	0	3 (14.3)	0	0	0	4 (5.5)	0
Gait disturbance	0	0	0	0	1 (12.5)	0	1 (4.8)	0	1 (14.3)	1 (14.3)	3 (4.1)	1 (1.4)
Nervous system disorders	1 (100)	0	8 (22.2)	0	3 (37.5)	0	11 (52.4)	2 (9.5)	6 (85.7)	0	29 (39.7)	2 (2.7)
Neuropathy peripheral	0	0	2 (5.6)	0	3 (37.5)	0	3 (14.3)	0	5 (71.4)	0	13 (17.8)	0
Headache	0	0	2 (5.6)	0	1 (12.5)	0	5 (23.8)	0	0	0	8 (11.0)	0
Dizziness	0	0	0	0	0	0	2 (9.5)	1 (4.8)	2 (28.6)	0	4 (5.5)	1 (1.4)
Lethargy	0	0	2 (5.6)	0	1 (12.5)	0	0	1 (4.8)	0	0	3 (4.1)	1 (1.4)
Paraesthesia	1 (100)	0	1 (2.8)	0	1 (12.5)	0	1 (4.8)	0	0	0	4 (5.5)	0
Peripheral sensory neuropathy	0	0	1 (2.8)	0	0	0	2 (9.5)	0	1 (14.3)	0	4 (5.5)	0
Vascular disorders	0	0	3 (8.3)	1 (2.8)	1 (12.5)	2 (25.0)	3 (14.3)	10 (47.6)	1 (14.3)	5 (71.4)	8 (11.0)	18 (24.7)
Hypertension	0	0	2 (5.6)	1 (2.8)	1 (12.5)	2 (25.0)	2 (9.5)	10 (47.6)	1 (14.3)	5 (71.4)	6 (8.2)	18 (24.7)
Metabolism and nutrition disorders	0	0	4 (11.1)	0	1 (12.5)	0	7 (33.3)	1 (4.8)	1 (14.3)	0	13 (17.8)	1 (1.4)
Decreased appetite	0	0	4 (11.1)	0	0	0	8 (38.1)	0	1 (14.3)	0	13 (17.8)	0
Hypophosphataemia	0	0	0	0	0	0	0	1 (4.8)	0	0	0	1 (1.4)
Musculoskeletal and connective tissue disorders	0	0	0	0	1 (12.5)	0	5 (23.8)	2 (9.5)	2 (28.6)	0	8 (11.0)	2 (2.7)
Back pain	0	0	0	0	0	0	4 (19.0)	1 (4.8)	1 (14.3)	0	5 (6.8)	1 (1.4)
Investigations	0	0	8 (22.2)	0	0	0	0	1 (4.8)	0	0	8 (11.0)	1 (1.4)
Troponin T increased	0	0	1 (2.8)	0	0	0	0	1 (4.8)	0	0	1 (1.4)	1 (1.4)
Skin and subcutaneous tissue disorders	1 (100)	0	4 (11.1)	0	1 (12.5)	0	3 (14.3)	0	0	0	9 (12.3)	0
Rash	0	0	3 (8.3)	0	0	0	1 (4.8)	0	0	0	4 (5.5)	0
Blood and lymphatic system disorders	0	0	6 (16.7)	0	0	0	2 (9.5)	0	0	0	8 (11.0)	0
Anaemia	0	0	6 (16.7)	0	0	0	1 (4.8)	0	0	0	7 (9.6)	0
Neoplasms benign, malignant and unspecified (incl. cysts and polyps)	0	0	0	0	2 (25.0)	0	1 (4.8)	1 (4.8)	0	0	3 (4.1)	1 (1.4)
Tumour pain	0	0	0	0	2 (25.0)	0	1 (4.8)	1 (4.8)	0	0	3 (4.1)	1 (1.4)
Psychiatric disorders	0	0	0	0	0	0	3 (14.3)	1 (4.8)	0	0	3 (4.1)	1 (1.4)
Hallucinations and nightmare	0	0	0	0	0	0	0	1 (4.8)	0	0	0	1 (1.4)
Respiratory, thoracic and mediastinal disorders	0	0	1 (2.8)	0	0	0	2 (9.5)	1 (4.8)	0	0	3 (4.1)	1 (1.4)
Dyspnoea	0	0	1 (2.8)	0	0	0	2 (9.5)	1 (4.8)	0	0	3 (4.1)	1 (1.4)
Cardiac disorders	0	0	1 (2.8)	0	0	1 (12.5)	2 (9.5)	1 (4.8)	0	0	3 (4.1)	2 (2.7)
Myocardial infarction	0	0	0	0	0	1 (12.5)	0	0	0	0	0	1 (1.4)
Myocarditis	0	0	0	0	0	0	0	1 (4.8)	0	0	0	1 (1.4)

A patient with multiple events within a Preferred Term (PT) is counted only once in the PT (using the worst CTCAE grade).

Serious AEs were reported in 36 (49.3%) patients. Sixteen SAEs were considered possibly or probably treatment-related in 13 (17.8%) patients. Pyrexia (*n* = 3), tumour pain (*n* = 3), hypertension (*n* = 2), and acute coronary syndrome/myocardial infarction (*n* = 2) were the only treatment-related SAEs to occur in more than one patient.

Thirteen (17.8%) patients discontinued due to AEs including five (6.8%) patients for whom the AE was considered to be treatment related. Only one AE led to study drug discontinuation in more than one patient, intestinal obstruction which occurred in three (4.1%) patients treated at 30, 60, and 80 mg/m^2^, none of which were judged to be related to the study drug. Overall, 22 (30.1%) patients experienced treatment-emergent AEs that led to dose interruptions or modifications.

There was no apparent difference in the proportion of patients experiencing AEs when stratified by prior MTA exposure or not, and no significant odds ratio observed for any of the treatment-emergent AEs between the groups (data not shown).

### Pharmacokinetics

**Fig. 1:**
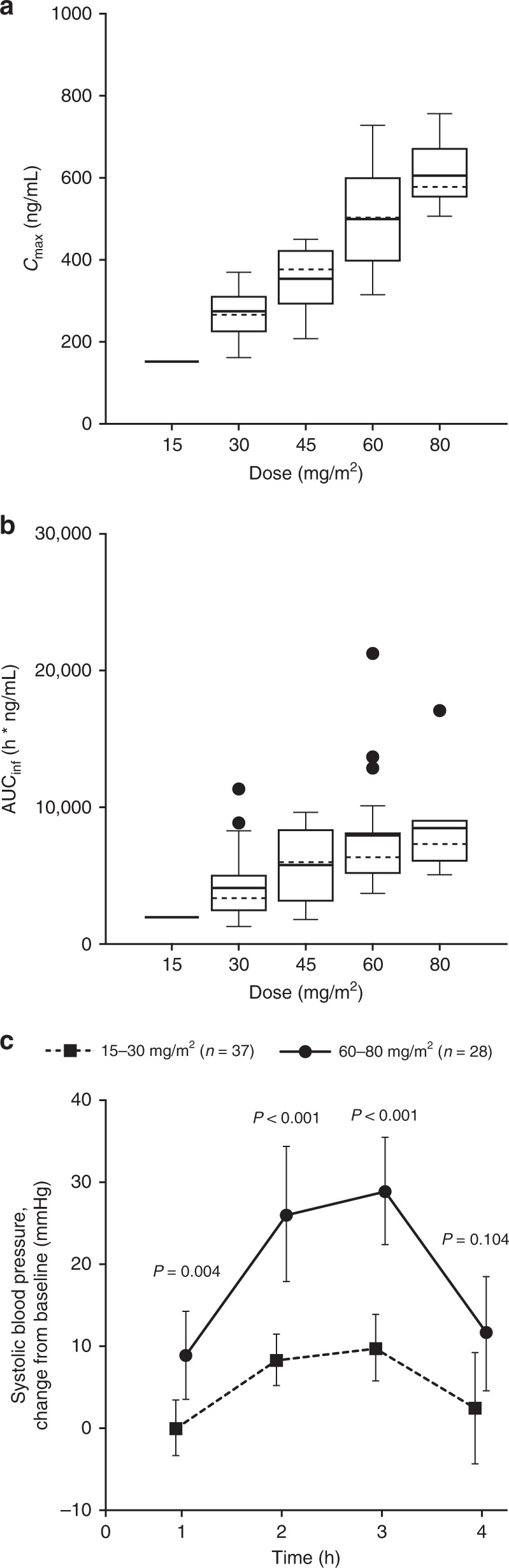
Pharmacokinetic profile of BAL27862 following administration of the prodrug BAL101553. BAL27862 exposure (**a** *C*_max_ and **b** AUC_inf_) and **c** mean change from baseline in systolic blood pressure according to BAL101553 dose at cycle 1, day 1 (*N* = 73). Box plots in (**a**, **b**) show median (solid horizontal line), mean (dashed horizontal line), interquartile range (box), 1.5 times the interquartile range (whiskers), and outliers (circles). Error bars in (**c**) indicate 95% confidence intervals.

Seventy-three patients were evaluable for PK on cycle 1, day 1, and 21 patients on cycle 2, day 1. The low number of evaluable patients on cycle 2, day 1 was due to patient discontinuations during cycle 1 due to either progressive disease or adverse events. Overall, the exposure of BAL101553 and BAL27862 was dose related (Fig. 1a, b, Supplementary Fig. 1A, B) and similar between male and female patients (Supplementary Fig. 2A, B).

The prodrug BAL101553 was rapidly converted to BAL27862 in all subjects and at all doses. In the RP2D (30 mg/m^2^) cohort, the BAL101553 prodrug had a moderate plasma clearance (CL: geometric mean 19.3 L/h) and low volume of distribution (*V*_ss_: 20.1 L), resulting in a short half-life (1.96 h). The active drug BAL27862 had a low apparent clearance (CL/*F*: 10.1 L/h) and moderate apparent volume of distribution (*V*_z_/*F*: 182 L), resulting in a half-life of 12.6 h.

Urinary excretion was not a significant route of elimination with <0.1% of the administered dose of BAL101553 and <1% of the equivalent dose for BAL27862 recovered in the urine (Supplementary Table 3). The PK parameters for BAL101553 and BAL27862 on day 1, cycle 1 are shown in Table 3 and Supplementary Table 4. The curves for all cohorts and PK days are shown in Supplementary Figs. 2A, B, 3.

**Table 3:** Summary of BAL27862 PK parameters for cycle 1, day 1 across dose cohorts.

Arm	*T*_max_ (h)	*C*_max_ (ng/mL)	*C*_max_/dose (ng/mL/mg)	AUC_last_ (h × ng/mL)	*T*_1/2_ (h)	AUC_inf_ (h × ng/mL)	CL/*F* (mL/h)	*V*_*z*_/*F* (mL)
15 mg/m^2^								
*N* Obs	1	1	1	1	1	1	1	1
Mean	2.5	154	9.62	1810	18.1	2110	7610	199,000
Geometric mean	2.5	154	9.62	1810	18.1	2110	7610	199,000
CV% geometric mean	—	—	—	—	—	—	—	—
30 mg/m^2^								
*N* Obs	36	36	36	36	36	36	36	36
Mean	2.17	271	7.5	2680	14.4	4150	11,500	200,000
Geometric mean	2.13	267	7.3	2530	12.6	3620	10,100	183,000
CV% geometric mean	16.7	19.8	24.1	35.3	57.2	55.7	58.7	41.3
45 mg/m^2^								
*N* Obs	8	8	8	8	8	8	8	8
Mean	2.39	356	7.02	4340	13.6	5770	12,000	190,000
Geometric mean	2.3	346	6.8	3960	12.5	5090	10,000	180,000
CV% geometric mean	28.8	26.3	29.2	51.9	47.7	62.9	67	33.7
60 mg/m^2^								
*N* Obs	21	21	21	21	21	21	21	21
Mean	2.41	498	6.98	6040	14.3	7920	10,600	206,000
Geometric mean	2.34	484	6.79	5580	13.8	7180	9910	197,000
CV% geometric mean	23.8	25.1	25	39.7	26.5	44.5	41	32.8
80 mg/m^2^								
*N* Obs	7	7	7	7	7	7	7	7
Mean	2.35	606	6.28	7760	12.9	8560	12,700	227,000
Geometric mean	2.33	601	6.25	7330	12.5	7950	12,100	218,000
CV% geometric mean	14.3	13.1	11	36.2	26.7	40.5	35.8	31.2

*AUC*_*inf*_ area under the curve from time zero to infinity, *AUC*_*last*_ area under the curve from time zero to last measurable concentration, *CL/F* apparent clearance, *C*_*max*_ maximum concentration, *CV%* percentage of coefficient of variation, *T*_*1/2*_ terminal half-life, *T*_*max*_ time taken to reach *C*_*max*_, *V*_*z*_*/F* apparent volume of distribution in terminal phase.

#### Pharmacokinetic/adverse reaction relationships

A dose relationship was observed for nausea/vomiting, transient arterial hypertension, peripheral sensory neuropathy, and pain at tumour site. Arterial hypertension was significantly higher in patients treated with 60–80 mg/m^2^ BAL101553 than at 15–30 mg/m^2^ (Fig. 1c) and subsided over the course of several hours after the end of the 2-h intravenous study-drug administration. Peak blood pressure elevations occurred when the concentrations of the active drug BAL27862 were highest (during the first hours after the end of the 2-h infusion).

### Pharmacodynamics

Functional MRI was conducted in eight (11.0%) patients, six of whom had complete datasets and were evaluable (five treated at 30 mg/m^2^ and one at 45 mg/m^2^). There was no significant difference in cohort mean values for *K*^trans^ (volume transfer constant; *p* = 0.95), IAUGC_60_ (the integrated area under the gadolinium curve in tissue over 60 s; *p* = 0.93) and the Apparent Diffusion Coefficient (*p* = 0.92) across the five imaging time points, suggesting no observable treatment effect on dynamic contrast-enhanced MRI and diffusion-weighted MRI parameters within this cohort.

Twenty-four patients provided 1–3 pre- and post-treatment biopsies (median = 2). Evaluable pairs of pre- and post-treatment biopsies were available from six patients. Reductions in tumour microvascular density (five patients, based on CD34 staining) and/or decreases in tumour cell proliferation (two patients, based on Ki67 staining) were apparent (Supplementary Table 5 and Supplementary Figs. 4, 5). There was no clear dose effect regarding the change from baseline in enumerations of circulating tumour cells, circulating endothelial cells, or circulating endothelial progenitor cells.

### Efficacy

**Fig. 2:**
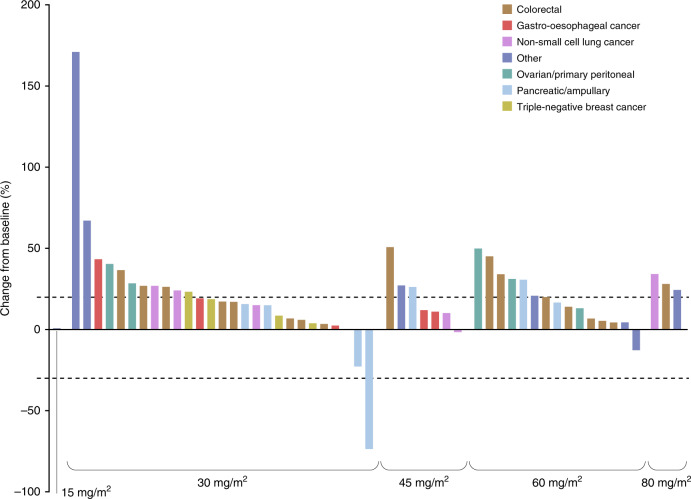
Waterfall plot of best percentage change from baseline in sum of longest diameter for target lesions (*N* = 53 evaluable patients). Upper dotted line indicates the RECIST v1.1 criteria for progressive disease; lower dotted line indicates the RECIST v1.1 criteria for partial response.

Overall, 57 (78.1%) patients were evaluable for efficacy. One patient with an ampullary carcinoma had a PR (starting dose level of 30 mg/m^2^ with subsequent dose escalation to 45 mg/m^2^). This PR lasted for over 2 years. The ORR was therefore 1.8% (95% CI: 0.0, 9.4). Fourteen (24.6%) other patients had SD lasting two or more cycles (median 15 weeks; range 7–33) as the best response (including four with SD lasting for four or more cycles), leading to an overall DCR of 26.3% (95% CI: 15.5, 39.7; *n* = 15). The overall median PFS was 50.0 days (95% CI: 50.0, 51.0). The PR, SD, and progressive disease rates in patients who received BAL101553 at the recommended phase 2 dose of 30 mg/m^2^ (*n* = 36) were 3.2%, 16.1%, and 80.6%, respectively, equating to an ORR of 3.2% (95% CI: 0.1, 16.7) and a DCR of 19.4% (95% CI: 7.5, 37.5) in these patients. There was no apparent relationship between tumour type and response. Overall, four of 53 (7.5%) evaluable patients had reductions in the sum of longest diameter from baseline for target lesions at least once during the study. These comprised the patient with ampullary carcinoma (target lesion: mesenteric lymph node; −73.3% change) and a patient with pancreatic cancer (two target lesions: liver, −22.6% change) treated at 30 mg/m^2^; a patient with NSCLC (two target lesions: lung, −1.3% change) treated at 45 mg/m^2^; and a patient with cervical cancer (target lesion: hilar lymph node, −12.5% change) treated at 60 mg/m^2^. The overall median maximum change in the sum of longest diameter from baseline was 17.9% (range −73.3 to 171%; Fig. 2). There was no clear difference in efficacy between patients with/without prior MTA treatment. The patient with ampullary carcinoma who had a PR had not had prior MTA treatment.

## Discussion

This was a two-part open-label study in patients with solid tumours designed to determine the MTD and DLT of a 2-h infusion of BAL101553, the lysine prodrug of BAL27862, and to investigate the overall safety tolerability, PK profile, and anti-tumour activity. The primary objective of the study was met by establishing the MTD for BAL101553 as initially 60 mg/m^2^ when administered intravenously over 2 h as single agent on days 1, 8, and 15 of a 28-day treatment cycle. The RP2D was determined as 30 mg/m^2^.

The DLTs were reversible Grade 2–3 gait disturbance at dose levels ≥60 mg/m^2^, occurring with Grade 2 peripheral sensory neuropathy and asymptomatic myocardial injury at dose levels ≥45 mg/m^2^. Most of the Grade 3 neurological events and all cardiac events occurred during or after the first infusion of study drug and appeared to be related to the *C*_max_ of BAL101553/BAL27862.

Peripheral neuropathy is common with MTA therapy due to their axonal microtubule disrupting activity.^2,5,22^ In this study, Grade 2 peripheral sensory neuropathy with reduced proprioception/sensation was observed in two patients treated with 80 mg/m^2^ BAL101553 and contributed to the DLTs of Grade 2–3 gait disturbance in these patients. At the RP2D (30 mg/m^2^), the incidence of Grade 1/2 peripheral neuropathy was relatively low (5.6%) and reversible, and no instances of Grade 3/4 peripheral neuropathy were reported. Furthermore, the incidences of other nervous system disorders commonly associated with MTA therapy, such as headache and dizziness,^3^ were low following treatment with BAL101553.

Cardiovascular toxicity was not seen in standard non-clinical toxicity studies including a telemetered dog study. However, several study-design changes were required to reflect the dose-related cardiac and vascular effects observed during the study and non-clinical data which arose while the study was ongoing that indicated a dose-related vascular-disrupting effect of BAL101553 in animal models. The changes to the protocol resulted in lower doses of BAL101553 being investigated in the phase 2a part of the study. The dose in the MTD-arm was reduced to 45 mg/m^2^ and then to 30 mg/m^2^, following the observation of clinical cardiovascular toxicity in three patients. The three instances of asymptomatic myocardial injury at dose levels ≥45 mg/m^2^ were interpreted as microvascular myocardial damage due to the vascular-disrupting properties of BAL101553/BAL27862. The incidence and the time course of blood pressure elevations provide further evidence for a *C*_max_-related vascular-disrupting effect of BAL101553. Rises in blood pressure predominantly occurred at dose levels ≥45 mg/m^2^. These elevations were transient and generally resolved within 24 h following the intravenous dose. Blood pressure elevations were uncommon in patients treated at doses of 15–30 mg/m^2^. The early occurrence of troponin elevations (peak levels were observed at 8 h after the end of infusion) is also consistent with a *C*_max_-related vascular effect, as is the reduction in the number of tumour microvessels in paired (pre- vs. post-dose) tumour biopsies. BAL101553 appears to be inducing significant microvascular changes, which may also explain the increasing occurrence of certain AEs such as hypertension with increasing dose.

Systemic side effects of BAL101553 that showed a clear dose relationship included nausea/vomiting, transient arterial hypertension, peripheral sensory neuropathy, gait disturbance, myocardial ischaemia/infarction, and possibly abdominal/tumour-related pain. These side effects were absent or clinically not relevant at dose levels of up to 30 mg/m^2^ but occurred with a low prevalence at the dose level of 45 mg/m^2^ and a high prevalence at the dose levels of 60 and 80 mg/m^2^. Events observed at the higher dose levels usually required clinical monitoring, additional diagnostic measures, and occasionally, dose reductions or study drug discontinuation (for peripheral neuropathy, gait disturbance, and myocardial injury) and therapeutic interventions (including antiemetic, antihypertensive treatment, analgesic treatment, or cardiac treatment equivalent to the treatment of an acute coronary syndrome). Additional systemic side effects considered characteristic of BAL101553, but which did not show a clear dose relationship, included anorexia, diarrhoea, fatigue, and pyrexia.

The incidence of gastrointestinal disturbances with BAL101553, namely nausea, vomiting and diarrhoea, and fatigue, was in keeping with those of other MTAs, particularly at doses above 30 mg/m^2^.^3^ Myeloid toxicity, particularly neutropenia, is a common and often severe side effect observed in MTA-based combination regimens.^2^ No AEs of neutropaenia were reported with BAL101553 in this study. The only blood/lymphatic system disorder observed was Grade 1−2 anaemia, occurring at a very low incidence (9.6%) and appearing to be unrelated to BAL101553 dose.

Overall, the RP2D of 30 mg/m^2^ determined in this study was well tolerated and showed signals of anti-tumour activity. The only Grade 3 drug-related toxicities at this level were fatigue and rare cases of transient hypertension (in patients with pre-existing hypertension). No relevant, drug-related neurological or cardiac toxicities were observed and there were only small effects on blood pressure. At this dose, with the largest number of patients (*n* = 36 on cycle 1, day 1), PK exposure showed low to moderate variability with geometric mean CV% of <20% for *C*_max_ and <60% for AUC_inf_.

The single PR in the patient with an ampullary carcinoma treated at 30 mg/m^2^ and escalated to 45 mg/m^2^ was long lasting. The PR response occurred at the starting dose level of 30 mg/m^2^ and this patient remained on treatment for 36 cycles before discontinuing due to the development of an allergic reaction to the study drug.

The 2-h intravenous dosing strategy is no longer being pursued; however, further investigation of BAL101553 is underway, exploring alternative dosing strategies to minimise the *C*_max_-related vascular toxicity, including oral dosing (EudraCT 2014-003371-34, NCT02490800) and administration as a 48-h continuous infusion (NCT02895360^23^). Encouraging data have been reported on the use of BAL101553 in combination with radiotherapy in paclitaxel and epothilone-resistant^14^ and orthotopic glioblastoma^19^ tumour models. A study of BAL101553 in combination with radiation therapy in patients with newly diagnosed glioblastoma (NCT03250299) is currently underway, and further investigations in patients with ovarian cancer and in patients with recurrent glioblastoma are planned.

In conclusion, BAL101553 is a novel spindle assembly checkpoint controller with both anti-proliferative and vascular-disrupting effects. BAL101553 given weekly as a 2-h infusion exhibited dose-limiting neurological and cardiovascular effects at dose levels ≥45 mg/m^2^ that appear to be related to maximum plasma concentration. The recommended phase 2 dose of 30 mg/m^2^ was well tolerated and showed preliminary signals of anti-tumour activity.

## Supplementary information

Supplementary Materials

## Data Availability

The study data for this trial are considered commercially proprietary and are not available for unrestricted access. All the authors had full access to all the data in the study and take responsibility for the integrity of the data and the accuracy of the data analysis.
