# Supplementary material for: Phase 1/2a trial of intravenous BAL101553, a novel controller of the spindle assembly checkpoint, in advanced solid tumours
Source: Br J Cancer. 2020 Aug 3;123(9):1360–9. doi: 10.1038/s41416-020-1010-8 (PMC7591872; doi:10.1038/s41416-020-1010-8)

# Phase 1/2a trial of intravenous BAL101553, a novel controller of the spindle assembly checkpoint, in advanced solid tumours

Rebecca Kristeleit, Jeffry Evans, L. Rhoda Molife, Nina Tunariu, Heather Shaw, Sarah Slater, Noor R Md Haris, Nicholas F Brown, Martin D Forster, Nikolaos Diamantis, Robert Rulach, Alastair Greystoke, Uzma Asghar, Mihaela Rata, Stephanie Anderson, Felix Bachmann, Alison Hannah, Thomas Kaindl, Heidi A. Lane, Patrice Larger, Anne Schmitt-Hoffmann, Marc Engelhardt, Alexandar Tzankov, Ruth Plummer, Juanita Lopez

***Supplementary data***

**Supplemental methods**

Supplemental method section containing details of the randomisation period introduced during Phase 2a of the study ([Supplementary text 1](#NewSText1)), full inclusion and exclusion criteria ([Supplementary text 2](#SText1)), and definitions of dose-limiting toxicity criteria ([Supplementary text 3](#SText2)).

**Supplementary tables**

- [Supplementary table 1](#STab1): Dose escalation criteria
- [Supplementary table 2](#NewSTab2): Baseline demographic and disease history data the 73 enrolled and treated patients by dose group and overall
- [Supplementary table 3](#STab2): 24-hour urinary excretion of BAL101553 and BAL27862
- [Supplementary table 4](#STab3): Overview of BAL101553 and BAL27862 PK parameters for Cycle 1, Day 1 across dose cohorts
- [Supplementary table 5](#STab4): Changes in proliferation (Ki67) and vascularization (CD34) markers in post-treatment vs pre-treatment biopsies.

**Supplementary figures**

- [Supplementary figure 1](#SFig1): Pharmacokinetics of BAL101553 and BAL27862 at Cycle 1, Day 1
- [Supplementary figure 2](#SFig2): Geometric mean plasma concentration-time of BAL101553 and BAL27862 at Cycle 1, Day 1 across sex groups (30 mg/m^2^ cohort)
- [Supplementary figure 3](#SFig3): Spaghetti plot of PK data by cohort and PK days: BAL101553 and BAL27862
- [Supplementary figure 4](#SFig4): Reduction in tumor vascularization and focal anti-proliferative effects in a post-treatment tumor biopsy (60 mg/m^2^ BAL101553; Day 22, Cycle 1)
- [Supplementary figure 5](#SFig5): Anti-proliferative effect observed in a post-treatment tumor biopsy (60 mg/m^2^ BAL101553; Day 22, Cycle 1)

Supplementary Text 1: Randomisation in Phase 2a

The Phase 2a expansion part was originally intended to characterise the safety, tolerability, and efficacy of BAL101553 at the maximum tolerated dose (MTD) level determined in the Phase 1 part (60 mg/m^2^).

The protocol for Phase 2a of the study was modified in several amendments to investigate whether a lower dose level was clinically preferable to dosing at the maximum tolerated dose. These changes were primarily based on the observation of dose-related vascular effects in Phase 1 of the trial, supported by preclinical data from other studies that emerged during the course of the trial which indicated a dose-related vascular-disrupting effect of BAL101553 in animal models.

A protocol amendment (Amendment F) was issued allowing patients to be randomised to receive either the MTD (60 mg/m^2^) or 50% of the maximum tolerated dose (30 mg/m^2^). The 60 mg/m^2^ dose was reduced to 45 mg/m^2^ (Amendment H) after two patients treated with 60 mg/m^2^ BAL101553 exhibited myocardial injury (modified randomisation).

The 45 mg/m^2^ dose was subsequently discontinued (Amendment J) after a patient treated at this level experienced asymptomatic myocardial infarction. All remaining patients in the Phase 2a part received a dose of 30 mg/m^2^ (i.e. not randomised).

**Number of patients treated in Phase 2a during the initial randomisation, modified randomisation, and non-randomised period (n=49)**

|  | **Dose (mg/m^2^)** | | | |
| --- | --- | --- | --- | --- |
|  | **30** | **45** | **60** | **Total** |
| Phase 2a: Initial randomisation | 12 | - | 11 | 23 |
| Phase 2a: Modified randomisation | 4 | 5 | - | 9 |
| Phase 2a: Non-randomised | 17 | - | - | 17 |
| **Total** | **33** | **5** | **11** | **49** |

During the randomised period of the study, randomisation of patients was done centrally at Basilea. An unblinded statistician generated a randomisation list and sealed envelopes containing the dose allocation and provided these to each participating study site. Block sizes were assigned randomly in a 1:1 ratio to be 6 or 4 every 10 numbers. To minimise potential bias, the block size was defined and documented by the unblinded statistician and kept confidential until database lock.

Once a patient had completed all screening procedures and was confirmed as eligible for dosing in the study, the investigator, pharmacist, or designee received a number from the sponsor which assigned a sealed envelope. Randomisation was balanced within each study site.

Supplementary Text 2: Inclusion and exclusion criteria

### Inclusion criteria

Patients were eligible for study enrolment if they met all of the following inclusion criteria at screening. Screening evaluations were performed within 14 days prior to receiving first study drug administration (within 35 days for radiology assessments).

1. Age 18 years or older.
2. Patients with advanced or recurrent solid tumour types (either histologically or cytologically confirmed), who had failed standard therapy or for whom no effective standard therapy is available. In the Phase 2a part this was limited to patients with the following cancer types:
   1. colorectal cancer
   2. gastric cancer or cancers of the gastro-oesophageal junction
   3. non-small cell lung cancer
   4. ovarian (or primary peritoneal) cancer
   5. pancreatic cancer (including ampullary cancer)
   6. triple-negative breast cancer.
      The initial pathological diagnosis of breast cancer or the first recurrence of breast cancer outside of bone was required to be oestrogen receptor and progesterone receptor negative (< 1% by immunohistochemistry) and HER2 receptor negative by institutional guidelines, typically 0 – 1+ by immunohistochemistry or non-amplified by in situ hybridisation with a gene ratio by dual probe of < 2.0 or < 4.0 gene copies/cell by single probe.
3. Patients with known brain metastases had to have had stable disease for at least 3 months prior to starting the study drug. For these patients, imaging of the brain was required during the 14-day screening period; patients had to have undergone definitive local therapy (resection and/or radiation).
4. Measurable disease (according to RECIST criteria v1.1) documented within 35 days prior to starting study drug, or non-measurable ovarian cancer that could be followed by CA-125 or prostate cancer that could be followed by PSA, documented within 14 days prior to starting study drug.
5. Life expectancy ≥ 12 weeks.
6. Acceptable organ and marrow function was documented within 14 days prior to starting study drug, defined as follows:
   1. leukocytes ≥ 3.0 x 10^9^/L
   2. absolute neutrophil count ≥ 1.5 x 10^9^/L
   3. platelets ≥ 100 x 10^9^/L
   4. haemoglobin ≥ 9 g/dL
   5. total bilirubin ≤ 1.5 x institutional ULN
   6. AST and ALT ≤ 2.5 x institutional ULN or ≤ 5 x ULN in presence of liver metastasis
   7. serum creatinine ≤ 1.5 x institutional ULN, or creatinine clearance ≥ 60 mL/min by Cockcroft-Gault formula.
7. Patients had to have an ECOG performance status ≤ 1.
8. Female patients who were not pregnant or breast-feeding and met one of the following conditions:
   1. postmenopausal for at least 1 year;
   2. post‑hysterectomy and/or post‑bilateral ovariectomy;
   3. women of childbearing potential had to have a negative serum human chorionic gonadotropin (hCG) pregnancy test and had to use adequate contraceptive methods including barrier contraception for the duration of the study and for an additional 30 days after the last dose of study drug.
9. Male patients had to agree not to donate sperm from the first dose of study drug until 90 days after the end of treatment. Male patients, without a vasectomy and with a partner of childbearing potential, had to agree to use condoms and be instructed that their female partner should use another form of contraception for the duration of the study and continue for 30 days after the last dose of study drug.
10. Signed, written informed consent had to be obtained and documented according to ICH-GCP, the local regulatory requirements, prior to study-specific screening procedures.

### Exclusion criteria

Patients were excluded from the study if any of the following criteria were met:

1. Patients who had received chemotherapy, radiotherapy, immunotherapy, or investigational agents within 4 weeks (2 weeks for oral chemotherapy, hormonal therapy, biologics, or palliative radiotherapy; 6 weeks for nitrosoureas or mitomycin C) prior to starting study drug; patients who had not recovered to CTCAE v4.03 Grade ≤ 1 from all side effects of prior therapies except for residual toxicities, such as alopecia, which do not pose an ongoing medical risk.
2. Symptomatic brain metastases (including leptomeningeal disease) indicative of active disease.
3. Peripheral neuropathy CTCAE v4.03 Grade ≥ 2.
4. Known human immunodeficiency virus infection.
5. Known acute or chronic hepatitis B or hepatitis C infection.
6. Confirmed clinic SBP ≥ 140 mmHg and/or DBP ≥ 90 mmHg observed as part of the screening examination. Patients with an initial clinic BP ≥ 140/90 mmHg may be included if SBP < 140 mmHg and DBP < 90 mmHg is confirmed in two subsequent BP measurements on the same day, or if subsequent daytime average from 24-hour ambulatory blood pressure monitoring is SBP < 130 mmHg and DBP < 85 mmHg.
7. Patients who had been treated with a calcium channel blocker or who required a combination of more than two antihypertensive medications to control blood pressure.
8. History of cerebral haemorrhage, cerebral aneurysm, ischemic stroke or transient ischemic attack.
9. Significant cardiac disease or abnormality, including any one of the following:
   1. left ventricular ejection fraction < 50% at screening (assessed by echocardiography)
   2. QTcF > 470 msec on screening ECG or a clinically relevant ECG abnormality
   3. congenital long QT syndrome
   4. history of sustained ventricular tachycardia, ventricular fibrillation or torsades de pointes
   5. presence of atrial fibrillation with tachyarrhythmia (ventricular response rate > 100 bpm)
   6. bradycardia (heart rate < 50 bpm)
   7. complete left bundle branch block
   8. bifascicular block (complete right bundle branch block and anterior or posterior left hemiblock)
   9. myocardial infarction, acute coronary syndrome (including unstable angina), coronary revascularisation procedures or peripheral/coronary arterial bypass grafting within 6 months prior to starting study drug
   10. cardiac troponin (either troponin T or troponin I) above institutional ULN
   11. congestive heart failure of New York Heart Association class III or IV.
10. Uncontrolled intercurrent illness that would unduly increase the risk of toxicity or limit compliance with study requirements in the opinion of the investigator; including but not limited to: ongoing or active symptomatic infection, uncontrolled diabetes mellitus, unstable or uncompensated cardiac, hepatic, renal, respiratory, or psychiatric illness.
11. Ongoing anticoagulation treatment, including treatment with warfarin potassium or other coumarin derivates. Heparin/low molecular weight heparin, aspirin, or other oral platelet inhibitors were permitted.
12. Women who were pregnant or breast-feeding. Men or women of reproductive potential who were not willing to apply effective birth control during the study and for at least 30 days after the last dose of study drug in both sexes.

Supplementary Text 3: Standard dose-limiting toxicity criteria

Dose escalation was performed using a modified accelerated titration design and cohorts were expanded if a patient experienced a DLT, defined as any one of the following BAL101553 treatment-related events, graded according to CTCAE v4.03, during the first 28-day treatment cycle:

- Grade 4 neutropenia (ANC < 0.5 x 10^9^/L) lasting for ≥ 5 consecutive days
- Febrile neutropenia (ANC < 1.0 x 10^9^/L and single temperature of > 38.3°C or a sustained temperature of ≥ 38.0°C for more than one hour)
- Grade 4 thrombocytopenia (platelet count < 25 x 10^9^/L) or Grade 3 thrombocytopenia (platelet count < 50 x 10^9^/L) with bleeding
- Grade ≥ 3 nausea, vomiting or diarrhoea despite appropriate pre-medication and/or management
- Grade 3 AST/SGOT or ALT/SGPT elevations (> 5 – 20 x ULN) for more than 7 days, or Grade 4 (> 20 x ULN) for any duration
- Grade 3 QTc interval prolongation (QTcF > 500 msec or > 60 msec change from baseline)
- Hypertension related DLT (see note below*):
- Grade 4 Hypertension (CTCAE v4.03)
- Any recording of systolic blood pressure (SBP) > 220 mmHg or diastolic blood pressure (DBP) > 110 mmHg
- At least one observation of SBP ≥ 160 mmHg or DBP ≥ 100 mmHg that does not resolve to SBP < 160 mmHg and DBP < 100 mmHg within 24 hours, despite antihypertensive treatment
  The need for administration of new antihypertensive medication or modification to more intensive antihypertensive medication was not considered to be a DLT
- Any adverse event leading to missing both the Day 8 and Day 15 doses in Cycle 1 or causing a delay in the start of Cycle 2 by more than 14 days
- Any other Grade ≥ 4 haematological or other Grade ≥ 3 non-haematological AE with the following exceptions:
- Grade 3 fatigue unless considered clinically significant
- Grade 3 or 4 elevations in alkaline phosphatase unless considered clinically significant
- Grade 4 lymphopenia unless considered clinically significant
- Any other AEs that, in the view of the investigator and the sponsor, represents a clinically significant hazard to the patient.

* In the original study protocol, any drug-related CTCAE v4.03 Grade ≥ 3 non-haematological AEs was considered to be a DLT. Under this definition, any confirmed blood pressure elevation of SBP ≥ 160 mmHg or DBP ≥ 100 mmHg (if worsened from baseline) considered at least possibly related to BAL101553 met a DLT criterion. This definition was considered over-sensitive for the transient blood pressure (BP) elevations observed with BAL101553 and other anti-neoplastic agents that fall into the class of vascular disrupting agents. Following the observation of asymptomatic transient Grade 3 hypertension in the first cycle of therapy in two patients treated at 60 mg/m^2^, the protocol was modified to safeguard the use of BAL101553 at dose levels above 30 mg/m^2^ and to allow re-exploration of higher dose levels using a modified DLT definition.

Supplementary Table 1: Dose escalation criteria

| **Modified accelerated titration Phase (provisional cohort size of one patient)** | | |
| --- | --- | --- |
| **Provisional dose cohorts** | **No. of pts with treatment-related DLT or CTCAE v4.03 Grade ≥ 2 event ^a^** | **Escalation decision** |
| Cohort 1 - Cohort 3 | None | Proceed to the next dose level (see Table 1). |
| Cohort 1 - Cohort 3 | First occurrence of a CTCAE Grade ≥ 2 (but non-DLT) event in any cohort | Expand current dose cohort to a total of 3 pts.  If no DLT in 3 pts, enrol 3 pts to next higher dose level (see Table 1) and continue with 3+3 Titration. Dose doubling will be maintained. |
| Cohort 1  (15 mg/m^2^ per week) | DLT in the first enrolled patient | Reduce to dose level ‑1 (7.5 mg/m^2^) and enrol 3 pts.  Continue with 3+3 Titration with approximately 40% dose increments for subsequent dose cohort escalation.  If DLT is observed in any patient at dose level ‑1, stop dosing, reduce to dose level ‑2 and enrol 3 pts. |
| Cohort 2 | 1 patient with DLT | Expand current dose cohort and continue with 3+3 Titration design with approximately 40% increments for subsequent dose cohort escalation. |
| Cohort 3 | 1 patient with DLT | Expand current dose cohort and continue with 3+3 Titration design with approximately 33% increments for subsequent dose cohort escalation. |
| **Standard 3+3 titration Phase (provisional cohort size of three patients)** | | |
| **Provisional dose cohorts** | **No. of pts with treatment-related DLT in the first 3 pts of a dose cohort ^a^** | **Escalation decision** |
| Cohort 4  and onwards | 0 of 3 pts with DLT | Enter 3 pts at the next dose level. |
|  | ≥ 2 of 3 pts with DLT | Dose escalation will be stopped. This dose level will be declared as the MAD. At least 3 additional pts will be entered at the next lower dose level if only 3 pts were treated previously at that dose. |
|  | 1 of 3 pts with DLT | Enter 3 additional pts at this dose level.  If 0 of these 3 pts (total of ≤ 1 of the 6 pts) experience DLT, enter 3 pts at the next higher dose level  If ≥ 1 of these 3 pts (total of ≥ 2 of the 6 pts) experience DLT, then dose escalation is stopped, and this dose is declared as MAD. 3 additional pts will be entered at the next lower dose level if only 3 pts were treated previously at that dose. |
| **Maximum administered dose (MAD):**  The MAD is the dose level with a rate of DLT in ≥ 33% of pts during treatment Cycle 1, i.e.:  ≥ 2 of [up to] 3 pts with DLT in the first 3 pts of a dose cohort.  ≥ 2 of [up to] 6 pts with DLT in a cohort that was expanded to 6 pts. | | |
| **Maximum tolerated dose (MTD):**  The MTD is the highest dose level below the MAD with an acceptable tolerability profile, i.e.:  Not more than 1 of 6 pts with DLT at the highest dose level below the MAD.  At least 6 patients must be treated at the MTD level during the dose escalation phase.  Intermediate dose levels may be assessed, e.g., if one dose is well tolerated without DLT and the subsequent dose level is defined as the MAD. | | |

^a^ Treatment related means causal relationship of the event to BAL101553 is considered to be at least “possible”.
The number of pts refers to pts evaluable for DLT assessment.
CTCAE v4.03, Common Terminology Criteria for Adverse Events version 4.0; DLT, dose-limiting toxicity; pts, patients; MAD, maximum administered dose; MTD, maximum tolerated dose.

Supplementary Table 2: Baseline demographic and disease history data the 73 enrolled and treated patients by dose group and overall

|  | BAL101553 15 mg/m^2^  (N=1) | BAL101553 30 mg/m^2^  (N=36) | BAL101553 45 mg/m^2^  (N=8) | BAL101553 60 mg/m^2^  (N=21) | BAL101553 80 mg/m^2^  (N=7) | BAL101553 Total  (N=73) | |
| --- | --- | --- | --- | --- | --- | --- | --- |
| Age (years), median (range) | 51.0 | 60.0 (32–79) | 67.0 (47–76) | 57.0 (29–80) | 55.0 (45–70) | 59.0 (29–80) | |
| Gender, n (%)  Female  Male | 1 (100)  0 | 16 (44.4)  20 (55.6) | 3 (37.5)  5 (62.5) | 11 (52.4)  10 (47.6) | 3 (42.9)  4 (57.1) | 34 (46.6)  39 (53.4) | |
| ECOG PS, n (%)  0  1 | 0 1 (100) | 10 (27.8)  26 (72.2) | 1 (12.5)  7 (87.5) | 9 (42.9)  12 (57.1) | 3 (42.9)  4 (57.1) | 23 (31.5)  50 (68.5) | |
| Prior treatment regimens, n (%)  Chemotherapy or hormone therapy  Radiotherapy  Surgery | 0  1 (100)  1 (100) | 36 (100)  13 (36.1)  26 (72.2) | 8 (100)  5 (62.5)  6 (75.0) | 21 (100)  7 (33.3)  16 (76.2) | 7 (100)  3 (42.9)  3 (42.9) | 72 (98.6)  29 (39.7)  52 (71.2) | |
| Most common tumor types, n (%)  Colorectal  NSCLC  Pancreatic/ampullary cancer  Gastro-esophageal cancer  Ovarian/primary peritoneal cancer  TNBC  Other* | 0  0  0  0  0  0  1 | 10 (27.8)  5 (13.9)  6 (16.7)  6 (16.7)  2 (5.6)  4 (11.1)  3 (8.3) | 1 (12.5)  2 (25.0)  1 (12.5)  2 (25.0)  1 (12.5)  0  1 (12.5) | 9 (42.9)  1 (4.8)  2 (9.5)  0  4 (19.0)  0  5 (23.8) | 2 (28.6)  1 (14.3)  0  0  1 (14.3)  0  3 (42.9) | 22 (30.1)  9 (12.3)  9 (12.3)  8 (11.0)  8 (11.0)  4 (5.5)  13 (17.8) | |
| Tumor histology, n (%)  Adenocarcinoma  Squamous cell carcinoma  Other | 0  0  1 (100) | 31 (86.1)  0  5 (13.9) | 6 (75.0)  2 (25.0)  0 | 18 (85.7)  1 (4.8)  2 (9.5) | 4 (57.1)  0  3 (42.9) | 59 (80.8)  3 (4.1)  11 (15.1) | |
| Metastatic disease, n (%) | 1 (100) | 34 (94.4) | 6 (75.0) | 18 (90.0) | 7 (100) | 66 (91.7) | |
| Histopathological grade, n (%)  Grade 1  Grade 2  Grade 3  Grade x (not assessable)  Other  Missing | 0  0  0  0  1 (100)  0 | 0  11 (30.6)  11 (30.6)  8 (22.2)  6 (16.7)  0 | 0  4 (50.0)  3 (37.5)  1 (12.5)  0  0 | 0  9 (45.0)  4 (20.0)  3 (15.0)  4 (20.0)  1 | 0  0  1 (16.7)  4 (66.7)  1 (16.7)  1 | 0  24 (33.8)  19 (26.8)  16 (22.5)  12 (16.9)  2 | |
| * Other comprises: two cases of esophageal cancer and single cases of adrenocortical cancer, anal cancer, cervical cancer, cholangiocellular cancer, epitheliod mesothelioma, gastric cancer, laryngeal cancer, mesenchymal chondrosarcoma, neuroendocrine cancer, small bowel cancer, thyoma  N = number of patients; NSCLC = non-small cell lung cancer; TNBC = Triple negative breast cancer; TNM = tumor, node, metastasis. | | | | | | |  |

Supplementary Table 3: 24-hour urinary excretion of BAL101553 and BAL27862

| **Visit** |  | **Analyte** | |
| --- | --- | --- | --- |
|  |  | **BAL27862** | **BAL101553** |
|  |  | **Amount recovered (% of dose)** | |
| Cycle 1 Day 1 | N | 21 | 20 |
|  | Mean | 0.174 | 0.0129 |
|  | Min | 0.0296 | 0.00297 |
|  | Max | 0.517 | 0.0228 |
|  | Geometric Mean | 0.144 | 0.0114 |
|  | CV% Geometric Mean | 73.0 | 58.4 |
| Cycle 2 Day 1 | N | 18 | 15 |
|  | Mean | 0.168 | 0.0123 |
|  | Min | 0.0636 | 0.00362 |
|  | Max | 0.385 | 0.0239 |
|  | Geometric Mean | 0.150 | 0.0109 |
|  | CV% Geometric Mean | 50.1 | 58.4 |

CV, coefficient of variation.

Supplementary Table 4: Overview of BAL101553 and BAL27862 PK parameters for Cycle 1, Day 1 across dose cohorts

|  |  | **BAL101553** | | | | | | | | **BAL27862** | | | | | | | |  |
| --- | --- | --- | --- | --- | --- | --- | --- | --- | --- | --- | --- | --- | --- | --- | --- | --- | --- | --- |
| Dose level | Parameter | N | Mean | SD | Min | Median | Max | Geometric Mean | CV% Geometric Mean | N | Mean | SD | Min | Median | Max | Geometric Mean | CV% Geometric Mean | |
| 15 mg/m^2^ | T_max_ (h) | 1 | 1.05 |  |  |  |  |  |  | 1 | **2.5** |  |  |  |  |  |  | |
|  | C_max_ (ng/mL) | 1 | 556 |  |  |  |  |  |  | 1 | **154** |  |  |  |  |  |  | |
|  | C_max_/Dose (ng/mL/mg) | 1 | 26 |  |  |  |  |  |  | 1 | **9.62** |  |  |  |  |  |  | |
|  | AUC_last_ (h*ng/mL) | 1 | 915 |  |  |  |  |  |  | 1 | **1810** |  |  |  |  |  |  | |
|  | T_1/2_ (h) | 1 | 0.908 |  |  |  |  |  |  | 1 | **18.1** |  |  |  |  |  |  | |
|  | AUC_inf_  (h*ng/mL) | 1 | 917 |  |  |  |  |  |  | 1 | **2110** |  |  |  |  |  |  | |
|  | AUC_inf_/Dose (h*ng/mL/mg) | 1 | 43 |  |  |  |  |  |  |  | **131** |  |  |  |  |  |  | |
|  | CL (L/h) | 1 | 23300 |  |  |  |  |  |  | 1 | **7610** |  |  |  |  |  |  | |
|  | CL (L/h/m^2^) | 1 | 14400 |  |  |  |  |  |  |  | **4700** |  |  |  |  |  |  | |
|  | V_ss_ (mL) | 1 | 11300 |  |  |  |  |  |  | 1 | **199000** |  |  |  |  |  |  | |
|  | V_ss_ (L/m^2^) | 1 | 6980 |  |  |  |  |  |  | 1 | **123000** |  |  |  |  |  |  | |
|  | MRT_inf_ (h) | 1 | 0.486 |  |  |  |  |  |  | 1 | **21.9** |  |  |  |  |  |  | |
| 30 mg/m^2^ | T_max_ (h) | 36 | 1.96 | 0.244 | 1 | 2 | 2.28 | 1.94 | 15.9 | 36 | 2.17 | 0.47 | 1.92 | 2.01 | 4.02 | 2.13 | 16.7 | |
|  | C_max_ (ng/mL) | 36 | 1170 | 500 | 262 | 1090 | 2220 | 1060 | 51.7 | 36 | 271 | 51.5 | 162 | 266 | 369 | 267 | 19.8 | |
|  | C_max_/Dose (ng/mL/mg) | 36 | 24.4 | 11.4 | 6.56 | 21.9 | 48.8 | 21.7 | 53.8 | 36 | 7.5 | 1.77 | 4.04 | 7.22 | 13.2 | 7.3 | 24.1 | |
|  | AUC_last_ (h*ng/mL) | 36 | 2430 | 1080 | 560 | 2210 | 4930 | 2170 | 53.3 | 36 | 2680 | 910 | 1290 | 2520 | 4430 | 2530 | 35.3 | |
|  | T_1/2_ (h) | 21 | 2.21 | 1.57 | 1.39 | 1.73 | 8.4 | 1.96 | 44.1 | 36 | 14.4 | 8.09 | 4.26 | 12 | 39.7 | 12.6 | 57.2 | |
|  | AUC_inf_  (h*ng/mL) | 21 | 2740 | 1100 | 999 | 2440 | 4930 | 2520 | 44 | 36 | 4150 | 2400 | 1370 | 3350 | 11400 | 3620 | 55.7 | |
|  | AUC_inf_/Dose (h*ng/mL/mg) | 21 | 57.2 | 26 | 20.3 | 47.6 | 105 | 51.8 | 48.7 | 36 | 116 | 74.3 | 37.3 | 92.3 | 410 | 99 | 58.7 | |
|  | CL (L/h) | 21 | 21400 | 9980 | 9480 | 21000 | 49200 | 19300 | 48.7 | 36 | 11500 | 5890 | 2440 | 10800 | 26800 | 10100 | 58.7 | |
|  | CL (L/h/m^2^) | 21 | 11400 | 5060 | 5330 | 10800 | 26300 | 10400 | 44 | 36 | 6160 | 2960 | 1730 | 5900 | 14400 | 5450 | 55.8 | |
|  | V_ss_ (mL) | 21 | 21900 | 9310 | 10300 | 21000 | 44300 | 20100 | 44.3 | 36 | 200000 | 98200 | 93900 | 174000 | 596000 | 183000 | 41.3 | |
|  | V_ss_ (L/m^2^) | 21 | 11700 | 4610 | 5780 | 10800 | 20600 | 10800 | 41.9 | 36 | 104000 | 38100 | 57600 | 97800 | 238000 | 98900 | 33.4 | |
|  | MRT_inf_ (h) | 21 | 1.07 | 0.249 | 0.523 | 1.08 | 1.43 | 1.04 | 28.2 | 36 | 20.2 | 11.4 | 7.31 | 16.8 | 55.8 | 17.7 | 55.8 | |

|  |  | **BAL101553** | | | | | | | | **BAL27862** | | | | | | | |  |
| --- | --- | --- | --- | --- | --- | --- | --- | --- | --- | --- | --- | --- | --- | --- | --- | --- | --- | --- |
| Dose level | Parameter | N | Mean | SD | Min | Median | Max | Geometric Mean | CV% Geometric Mean | N | Mean | SD | Min | Median | Max | Geometric Mean | CV% Geometric Mean | |
| 45 mg/m^2^ | T_max_ (h) | 8 | 1.91 | 0.694 | 1.03 | 2 | 3.25 | 1.8 | 39.6 | 8 | 2.39 | 0.813 | 1.97 | 2 | 4.22 | 2.3 | 28.8 | |
|  | C_max_ (ng/mL) | 8 | 1830 | 1030 | 23.3 | 1940 | 3430 | 1130 | 350 | 8 | 356 | 82.5 | 209 | 379 | 448 | 346 | 26.3 | |
|  | C_max_/Dose (ng/mL/mg) | 8 | 27 | 15.7 | 0.372 | 27.4 | 52.4 | 16.7 | 335 | 8 | 7.02 | 1.76 | 3.78 | 7.11 | 9.11 | 6.8 | 29.2 | |
|  | AUC_last_ (h*ng/mL) | 8 | 3570 | 2120 | 74.9 | 3690 | 7330 | 2320 | 262 | 8 | 4340 | 1850 | 1590 | 4360 | 7440 | 3960 | 51.9 | |
|  | T_1/2_ (h) | 8 | 1.57 | 0.307 | 1.15 | 1.62 | 1.9 | 1.54 | 20.7 | 8 | 13.6 | 5.72 | 6.54 | 14.1 | 22.8 | 12.5 | 47.7 | |
|  | AUC_inf_  (h*ng/mL) | 8 | 3570 | 2120 | 77.5 | 3690 | 7340 | 2340 | 257 | 8 | 5770 | 2750 | 1810 | 6070 | 9620 | 5090 | 62.9 | |
|  | AUC_inf_/Dose (h*ng/mL/mg) | 8 | 52.7 | 32.3 | 1.24 | 53 | 112 | 34.5 | 247 | 8 | 115 | 59.1 | 32.7 | 115 | 211 | 99.9 | 67 | |
|  | CL (L/h) | 8 | 118000 | 279000 | 8920 | 19100 | 808000 | 29000 | 247 | 8 | 12000 | 8530 | 4740 | 8700 | 30600 | 10000 | 67 | |
|  | CL (L/h/m^2^) | 8 | 73200 | 176000 | 5370 | 10700 | 508000 | 16900 | 257 | 8 | 6840 | 4550 | 3080 | 4880 | 16300 | 5830 | 63.1 | |
|  | V_ss_ (mL) | 8 | 348000 | 936000 | 9330 | 14500 | 2660000 | 28800 | 594 | 8 | 190000 | 75400 | 135000 | 161000 | 360000 | 180000 | 33.7 | |
|  | V_ss_ (L/m^2^) | 8 | 218000 | 589000 | 5310 | 8910 | 1680000 | 16700 | 630 | 8 | 109000 | 36100 | 83100 | 101000 | 193000 | 105000 | 28.3 | |
|  | MRT_inf_ (h) | 8 | 1.17 | 0.887 | 0.52 | 1.05 | 3.3 | 0.992 | 60.9 | 8 | 18.9 | 7.82 | 9.41 | 20.2 | 31.4 | 17.4 | 47.1 | |
| 60 mg/m^2^ | T_max_ (h) | 21 | 1.86 | 0.683 | 1.02 | 2 | 4.02 | 1.75 | 36.9 | 21 | 2.41 | 0.688 | 1.78 | 2.12 | 4.53 | 2.34 | 23.8 | |
|  | C_max_ (ng/mL) | 21 | 1740 | 923 | 30.3 | 1690 | 3640 | 1320 | 137 | 21 | 498 | 119 | 314 | 502 | 726 | 484 | 25.1 | |
|  | C_max_/Dose (ng/mL/mg) | 21 | 18.6 | 10 | 0.293 | 19.1 | 40.7 | 14 | 145 | 21 | 6.98 | 1.7 | 4.46 | 6.54 | 10.4 | 6.79 | 25 | |
|  | AUC_last_ (h*ng/mL) | 21 | 3190 | 1830 | 118 | 3340 | 7290 | 2510 | 109 | 21 | 6040 | 2980 | 2910 | 5190 | 17100 | 5580 | 39.7 | |
|  | T_1/2_ (h) | 16 | 1.59 | 0.491 | 0.833 | 1.59 | 2.63 | 1.51 | 33.1 | 19 | 14.3 | 4.34 | 10.2 | 12.6 | 27.5 | 13.8 | 26.5 | |
|  | AUC_inf_  (h*ng/mL) | 16 | 3780 | 1670 | 1250 | 3420 | 7310 | 3440 | 47.8 | 19 | 7920 | 4200 | 3810 | 6350 | 21300 | 7180 | 44.5 | |
|  | AUC_inf_/Dose (h*ng/mL/mg) | 16 | 40.2 | 18.4 | 11 | 36.6 | 79.9 | 36.2 | 52.7 | 19 | 110 | 53.7 | 50.8 | 93.8 | 283 | 101 | 41 | |
|  | CL (L/h) | 16 | 31400 | 19100 | 12500 | 27300 | 90900 | 27600 | 52.7 | 19 | 10600 | 3650 | 3540 | 10700 | 19700 | 9910 | 41 | |
|  | CL (L/h/m^2^) | 16 | 16900 | 8580 | 7190 | 15300 | 41900 | 15300 | 47.9 | 19 | 5920 | 2130 | 1850 | 6220 | 10400 | 5490 | 44.5 | |
|  | V_ss_ (mL) | 16 | 27300 | 30300 | 6260 | 19900 | 136000 | 20300 | 82 | 19 | 206000 | 61300 | 95200 | 198000 | 328000 | 197000 | 32.8 | |
|  | V_ss_ (L/m^2^) | 16 | 14500 | 14000 | 3700 | 10300 | 62500 | 11200 | 78 | 19 | 115000 | 36200 | 53000 | 107000 | 193000 | 109000 | 34.6 | |
|  | MRT_inf_ (h) | 16 | 0.836 | 0.396 | 0.208 | 0.902 | 1.49 | 0.733 | 61.5 | 19 | 19.3 | 6.92 | 10.8 | 17.7 | 40.5 | 18.3 | 31.7 | |

|  |  | **BAL101553** | | | | | | | | **BAL27862** | | | | | | | |  |
| --- | --- | --- | --- | --- | --- | --- | --- | --- | --- | --- | --- | --- | --- | --- | --- | --- | --- | --- |
| Dose level | Parameter | N | Mean | SD | Min | Median | Max | Geometric Mean | CV% Geometric Mean | N | Mean | SD | Min | Median | Max | Geometric Mean | CV% Geometric Mean | |
| 80 mg/m^2^ | T_max_ (h) | 7 | 1.61 | 0.472 | 1 | 1.97 | 2 | 1.54 | 32.9 | 7 | 2.35 | 0.327 | 1.97 | 2.55 | 2.68 | 2.33 | 14.3 | |
|  | C_max_ (ng/mL) | 7 | 3330 | 798 | 2440 | 3560 | 4450 | 3250 | 24.8 | 7 | 606 | 81.8 | 508 | 579 | 753 | 601 | 13.1 | |
|  | C_max_/Dose (ng/mL/mg) | 7 | 26.6 | 8.69 | 15.6 | 28 | 38.2 | 25.4 | 35.6 | 7 | 6.28 | 0.65 | 5.01 | 6.44 | 7.05 | 6.25 | 11 | |
|  | AUC_last_ (h*ng/mL) | 7 | 5920 | 1950 | 3610 | 7120 | 8200 | 5630 | 36.5 | 7 | 7760 | 3090 | 4890 | 6880 | 14100 | 7330 | 36.2 | |
|  | T_1/2_ (h) | 7 | 1.5 | 0.231 | 1.27 | 1.48 | 1.88 | 1.49 | 15.2 | 7 | 12.9 | 3.62 | 9.53 | 11.4 | 19.7 | 12.5 | 26.7 | |
|  | AUC_inf_  (h*ng/mL) | 7 | 5930 | 1950 | 3620 | 7140 | 8220 | 5640 | 36.5 | 7 | 8560 | 4050 | 5100 | 7330 | 17300 | 7950 | 40.5 | |
|  | AUC_inf_/Dose (h*ng/mL/mg) | 7 | 47.5 | 18.6 | 25.5 | 56.9 | 68 | 44 | 45.4 | 7 | 87 | 30.8 | 46.1 | 83.4 | 147 | 82.6 | 35.8 | |
|  | CL (L/h) | 7 | 24600 | 10800 | 14700 | 17600 | 39200 | 22700 | 45.4 | 7 | 12700 | 4560 | 6810 | 12000 | 21700 | 12100 | 35.8 | |
|  | CL (L/h/m^2^) | 7 | 13100 | 4690 | 8520 | 9800 | 19400 | 12400 | 36.5 | 7 | 7010 | 2290 | 3050 | 7180 | 10300 | 6620 | 40.5 | |
|  | V_ss_ (mL) | 7 | 14000 | 4950 | 9620 | 11500 | 22500 | 13300 | 34.6 | 7 | 227000 | 71200 | 144000 | 197000 | 349000 | 218000 | 31.2 | |
|  | V_ss_ (L/m^2^) | 7 | 7540 | 2070 | 5390 | 6920 | 10500 | 7300 | 27.4 | 7 | 125000 | 41600 | 79800 | 118000 | 172000 | 119000 | 35.1 | |
|  | MRT_inf_ (h) | 7 | 0.593 | 0.0833 | 0.465 | 0.574 | 0.719 | 0.588 | 14.3 | 7 | 16.5 | 5.48 | 12.1 | 14.9 | 27.7 | 15.8 | 30.1 | |

AUC_inf_, the area under the curve (AUC) extrapolated to infinity; AUC_last_, the area under the curve (AUC) from the time of dosing to the last measurable concentration; CL, clearance; C_max_, maximum observed concentration; CV, coefficient of variation; SD, standard deviation; T_1/2_, terminal half-life; T_max_, The time of maximum observed concentration; MRT_inf_, mean residence time extrapolated to infinity; V_ss_, volume of distribution at steady state.

Note: the names given in the Parameter column correspond to those for BAL101553, the intravenously administered prodrug; for the active BAL27862 the values given on the CL row actually are for CL/F, the apparent clearance corrected by the (undetermined) conversion factor (bioavailability) and the values given on the Vss rows actually are for Vz/F, the apparent volume of distribution during the terminal phase corrected by the (undetermined) conversion factor (bioavailability).

Supplementary Table 5: Changes in proliferation (Ki67) and vascularization (CD34) markers in post-treatment vs pre-treatment biopsies

| **Dose (mg/m^2^)** | **Primary** | **Biopsy Site** | **Marker** | |
| --- | --- | --- | --- | --- |
|  |  |  | **CD34** | **Ki67** |
| 60 | Peritoneal | Right inguinal lymph node | ↓ | ↓focal |
|  | Chondrosarcoma | Right cheek | ↑* | ↓↓ |
|  | Ovarian | Left inguinal lymph node | ↓ | - |
|  | Colorectal cancer | Lymph node | ↓** | - |
| 80 | Lung (neuroendocrine)^#^ | Paravertebral | ↓ | ↑ |
|  | Colorectal cancer | Abdomen | ↓ | ↑ |

Following immunohistochemical evaluation, proliferation was expressed as % Ki67 stained nuclei and tumour vascularization as the mean vessel density, using CD34 staining. Increased (↑) or decreased (↓) staining in the post‑treatment biopsy is indicated.

**↓** = moderate/faint change; ↓ = clear change; ↓↓= strong change; * = vascular staining much stronger in post-treatment sample; ** = limited tumour material for vascular evaluation; ^#^ = Post-treatment biopsy contained limited tumour material.

Supplementary Figure 1: Pharmacokinetics of (A) BAL101553 and (B) BAL27862 at Cycle 1, Day 1. For each figure part, the left and right charts show identical data (the left part uses a log Y-axis).


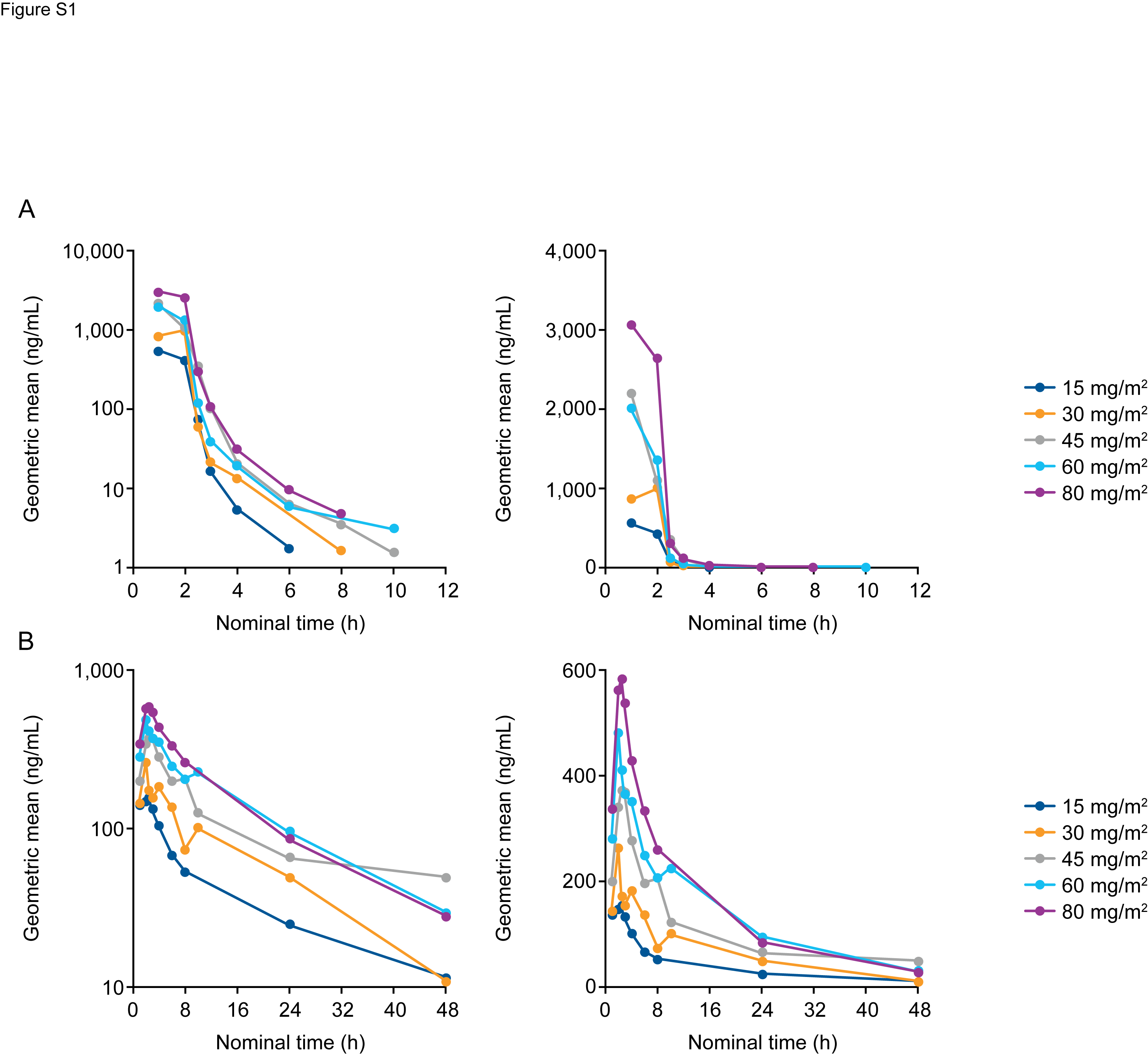


Supplementary Figure 2: Geometric mean plasma concentration-time of (A) BAL101553 and (B) BAL27862 at Cycle 1, Day 1 across sex groups (30 mg/m^2^ cohort).


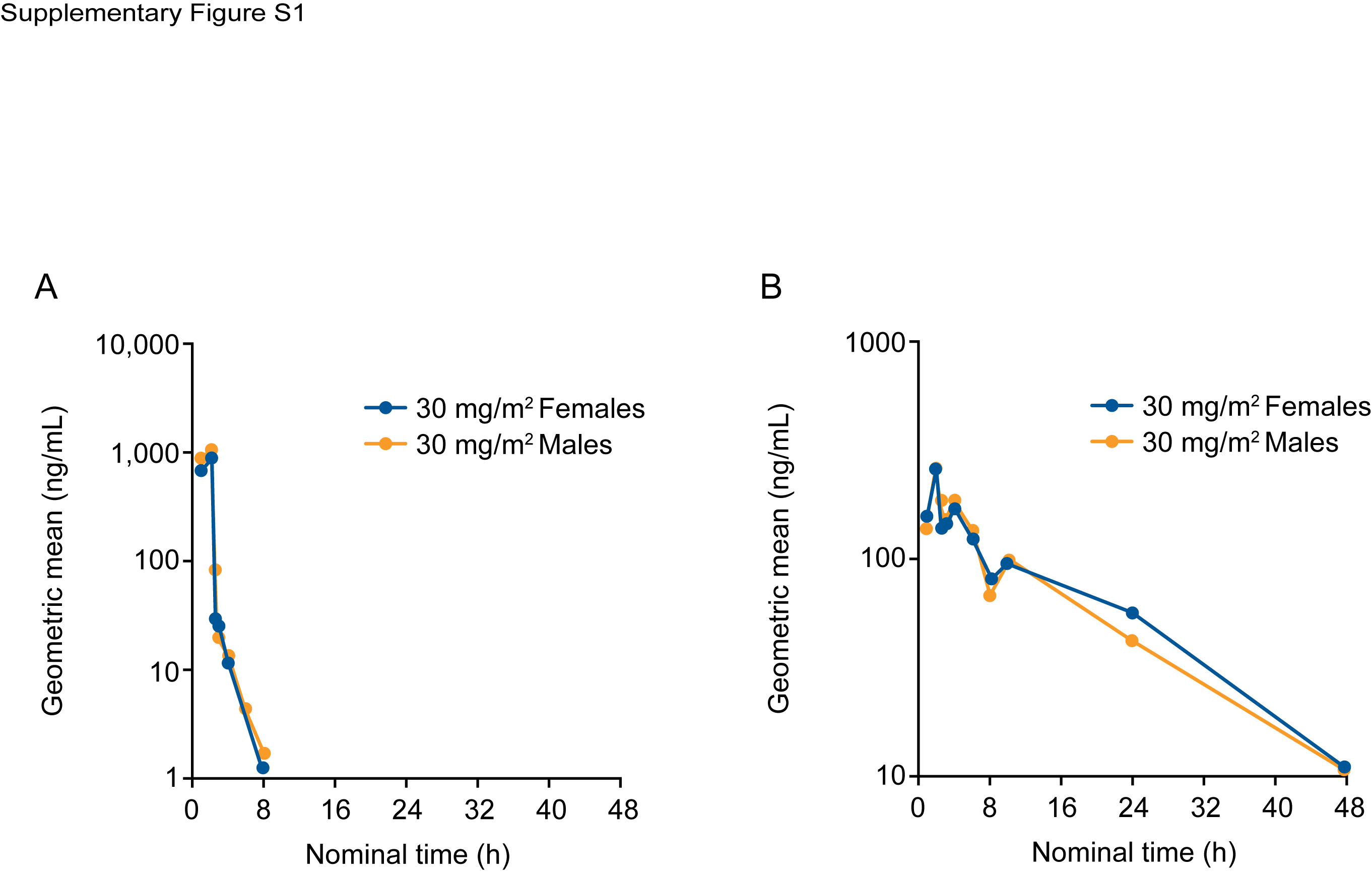


Supplementary Figure 3: Spaghetti plot of PK data by cohort and PK days: (A) BAL101553 and (B) BAL27862.


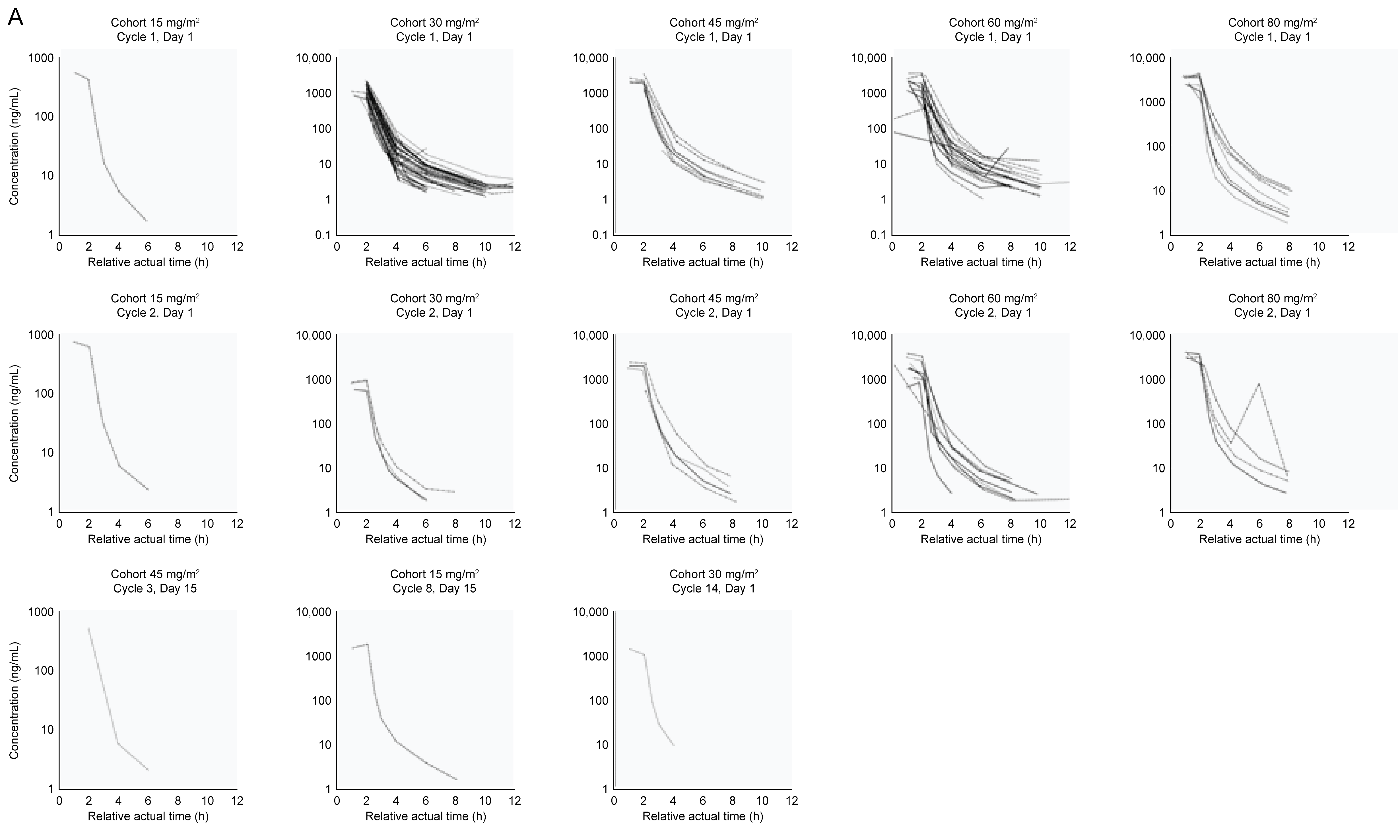


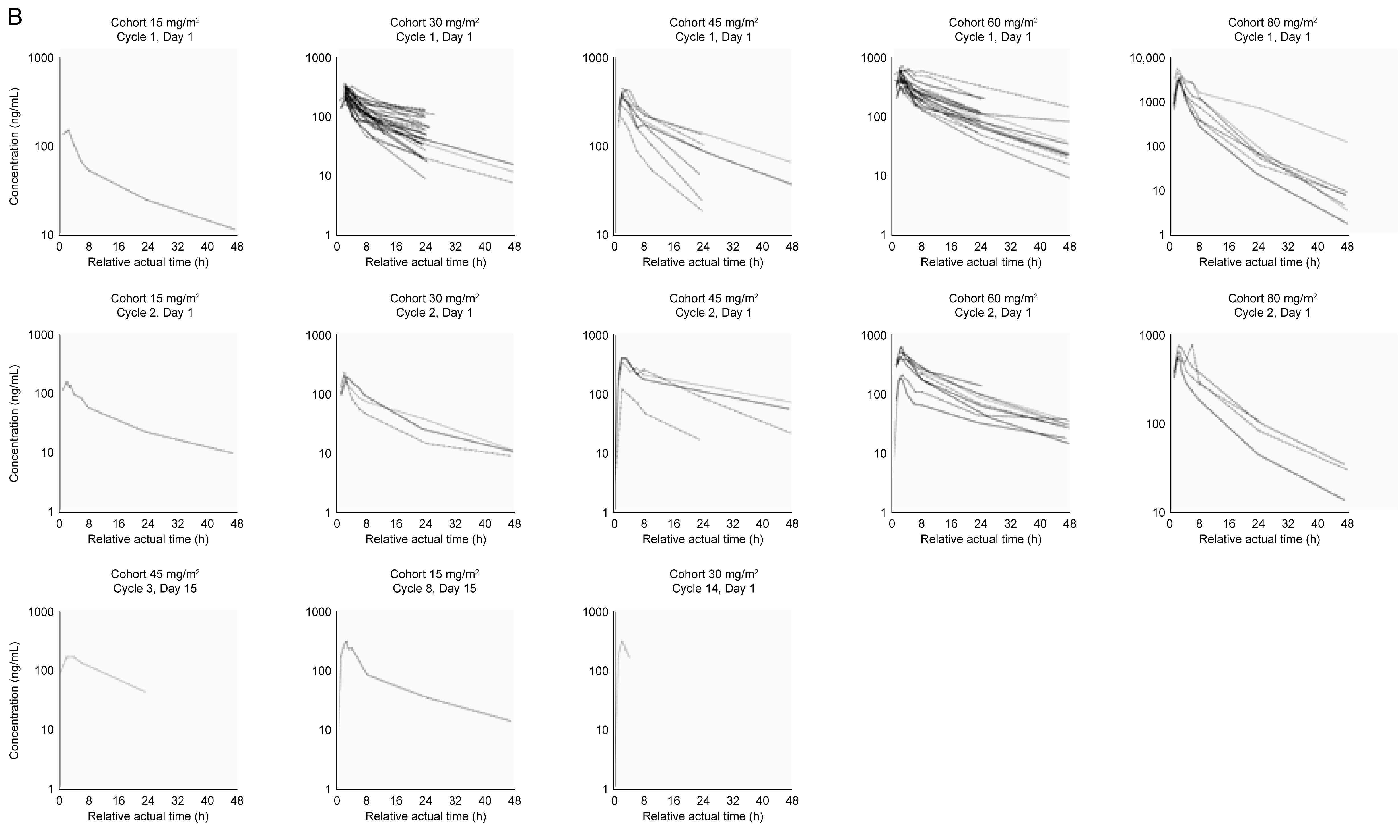


Supplementary Figure 4: Reduction in tumour vascularization and focal anti-proliferative effects in a post-treatment tumour biopsy (60 mg/m^2^ BAL101553; Day 22, Cycle 1).


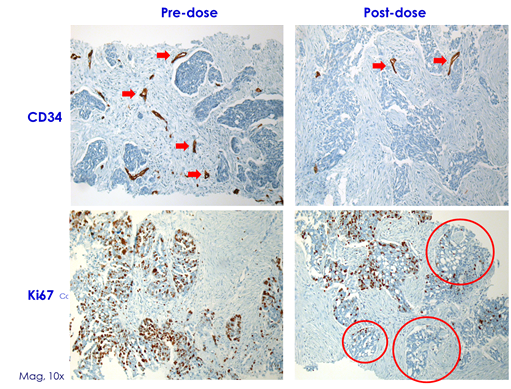


Arrows = CD34‑stained blood vessels; Circles = focal reduction of Ki67 staining of the nuclei of proliferating tumour cells.

Supplementary Figure 5: Anti-proliferative effect observed in a post-treatment tumour biopsy (60 mg/m^2^ BAL101553; Day 22, Cycle 1).


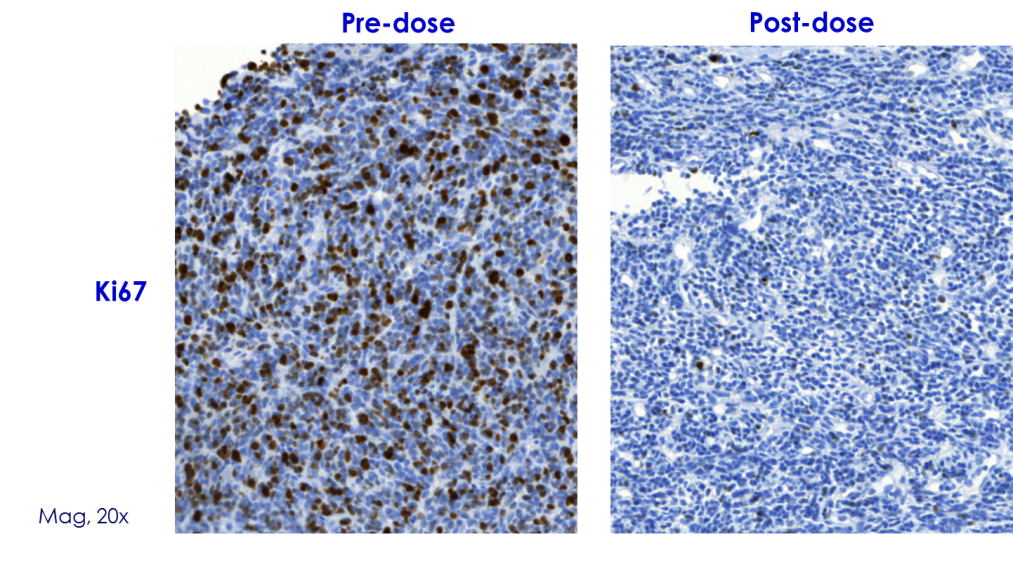

Supplement: Supplementary file 1 — Supplementary Materials [file 41416_2020_1010_MOESM1_ESM.docx]
